# Psychoneuroimmunological changes in adults with obesity receiving chiropractic care: a single-arm pilot trial

**DOI:** 10.3389/fpsyt.2026.1846987

**Published:** 2026-07-17

**Authors:** Tyson M. Perez, Margaret Sliwka, Daekiara Smith-Ireland, Amber Jackson, Stephanie Sullivan

**Affiliations:** 1 Life University, Marietta, GA, United States; 2 Peachy Keen Chiropractic, Marietta, GA, United States

**Keywords:** autonomic nervous system, chiropractic, obesity, patient reported outcome measures, pilot projects, psychoneuroimmunology

## Abstract

**Introduction:**

Psychoneuroimmunology (PNI) is an interdisciplinary framework that merges psychology, neurology, and immunology. Obesity, a condition of increasing global prevalence, is associated with elevated risk of PNI dysfunction. The primary aim of this single-arm pilot trial was to assess the feasibility of a PNI-focused assessment battery in combination with community-based chiropractic care in adults (18–65 years) with obesity. Additionally, we explored potential changes in PNI-related outcomes.

**Methods:**

Eighteen participants (12 females; mean age 48.7 ± 9.0 years) were enrolled and assigned to 6 weeks of chiropractic care. Assessments conducted at baseline, 2 weeks, and 6 weeks consisted of patient-reported outcomes (PROs), electrocardiography, impedance cardiography, and saliva collection via passive drool. Primary outcomes were eligibility, compliance, tolerability, adherence, and retention, with progression criteria based on the traffic light approach (i.e., red, amber, green). Secondary outcomes included changes in PRO scores, heart rate variability, pre-ejection period, and secretory immunoglobulin A.

**Results:**

Results indicated that our design is feasible with revisions. Although no causal inferences can be made, self-reported autonomic nervous system functioning and perceived stress levels demonstrated the largest within-group improvements among the PNI-related outcomes assessed.

**Discussion:**

Informed by these findings, we are planning a future definitive controlled trial to investigate the efficacy of chiropractic care on PNI-related outcomes in adults with obesity. This trial was prospectively registered on www.clinicaltrials.gov (NCT06208163) and funded by a seeding research grant from the Australian Spinal Research Foundation (SRG 2023–2 Perez).

## Introduction

Obesity has traditionally been defined as a body mass index (BMI) of ≥30 kg/m^2^ (1, 2). Per this definition, the global prevalence of obesity has doubled since 1980 (1) led by the U.S. with ~40% of adults meeting this criterion (2). However, newly published guidance suggests that, in the absence of extreme BMIs (i.e., >40 kg/m^2^) or direct body fat measurements (e.g., dual energy X-ray absorptiometry; DEXA), obesity can be presumed when at least one anthropometric criterion indicative of excess central adiposity (i.e., high waist circumference, waist-to-hip ratio, or waist-to-height ratio) is present (3). Remarkably, obesity prevalence in the U.S. adult population approaches 70% under these new guidelines (4).

Obesity is known to be associated with numerous psychoneuroimmunological (PNI)-related sequelae, including increased levels of psychological distress (5), cognitive deficits (6), dysautonomia (7), and immune system abnormalities (8). PNI is a relatively recent field of study which integrates the disciplines of psychology, neuroscience, and immunology to probe the interactions between thoughts, neurology, and immunity. Opposing a reductionist design that limits the observation of effects to a single paradigm, the PNI approach respects the multi-system evolutionary development of the organism (9).

The first key aspect of the PNI framework involves psychology. The psychological domain can be conceptualized as a combination of several interrelated components including mental health, emotional health, and beliefs & expectations (10). Commonly, psychological function is evaluated via the use of validated patient reported outcomes (PROs) such as the 10-item National Institutes of Health (NIH) Toolbox Perceived Stress Scale (PSS-10) (11).

The second integral aspect of the PNI paradigm is the neurological domain. Within this domain lies the autonomic nervous system (ANS). The ANS governs internal physiological processes such as heart rate and has two primary branches: the sympathetic and parasympathetic nervous systems (SNS and PSNS, respectively) (12). Generally, the PSNS is associated with “rest & digest” functions while the SNS is associated with a state of stress-induced “fight-or-flight” responses (13). There is a wide variety of validated procedures commonly employed to assess ANS functioning (14). For example, one of the most recognized surrogate measures of cardiac-related PSNS activity is respiratory-related heart rate variability (HRV; e.g., root mean square of successive difference, RMSSD) (15–18). Termed respiratory sinus arrhythmia, chronotropic (i.e., heart rate) changes occur during respiration due predominantly to the modulation of PSNS output at the sinoatrial node (15–18). As such, non-invasively tracking the heart’s *electrical* activity over many respiratory cycles via electrocardiography (ECG) allows researchers to estimate PSNS-mediated HRV, or more precisely heart period (i.e., interbeat interval; IBI) variability, over time. In contrast, systolic time intervals (e.g., pre-ejection period, PEP) are well-recognized and validated SNS measures which can be captured using non-invasive techniques such as impedance cardiography (ICG) (18). The SNS exerts inotropic (i.e., heart contraction) effects via the ventricular myocardium (18). ICG allows researchers to track the heart’s *mechanical* activity by monitoring electrical impedance changes in the thorax (19). Alternatively, neurological functioning can be subjectively assessed via validated PROs such as the 31-item Composite Autonomic Symptom Score (COMPASS-31) (20) and the 8-item Patient-Reported Outcomes Measurement Information System Cognitive Function Short Form (PROMIS Cog-8) (21).

The third and final PNI domain is immunology. The immune system’s primary role is in defense of the organism against a broad array of microbes and other potential pathogens (22). Immunoglobulins, or antibodies, are one of the key molecules synthesized by the immune system to protect against infections (23, 24). Although numerous immunoglobulins (i.e., IgA, IgD, IgE, IgG, IgM) can be measured across various body fluids, an increasing popular, non-invasive approach is the quantification of salivary immunoglobulins, most notably secretory immunoglobulin A (sIgA) (23, 24). Released by the mucosa, sIgA provides nonspecific, first-line defense against pathogens (23, 24).

While chiropractic research has often been focused on the pain-based results of care (25, 26), there is growing interest in exploring broader systemic impacts. Chiropractic is a profession founded on the premise that correction of vertebral subluxations via spinal adjustments restores proper function to the nervous system (27–30). The World Health Organization has defined a vertebral subluxation as “a lesion or dysfunction in a joint or motion segment in which alignment, movement integrity and/or physiological function are altered, although contact between joint surfaces remains intact” (31). Within the Sensory-Motor Integration theoretical framework, it has been hypothesized that vertebral subluxations impact the CNS via a breakdown in central segmental motor control that results in altered afferent feedback from paraspinal muscle spindles at the subluxated levels of the spine, potentially leading to upstream neural plastic changes in the CNS, and subsequent downstream aberrations in efferent output (32–34). However, these underlying mechanisms remain largely speculative.

While still preliminary, some functional magnetic resonance imaging (fMRI) studies (35, 36) and resting-state electroencephalography (EEG) trials (37–39) provide early data suggesting that chiropractic care may modulate CNS function. For example, a recent study suggested chiropractic care might modulate the activity of the prefrontal cortex (39), hinting at the potential for effects on other neurologically mediated processes such as executive functioning. Further, there are some limited data suggesting that chiropractic adjustments may modulate ANS activity (40, 41). In support of this possibility, a recent review concluded that “chiropractic manipulation [and] spinal manipulative therapy, which elicits sympathetic and parasympathetic responses, may improve autonomic balance” (40). With respect to the psychological domain, a recently published clinical trial on military service members suggests that chiropractic care may be associated with positive changes in various aspects of psychological functioning, including depression and anxiety (42). Finally, a few studies in relatively healthy populations have evaluated chiropractic’s potential impact on immune-related biomarkers such as salivary sIgA (43, 44). Taken together, these preliminary findings tentatively suggest that PNI-related dysfunctions (e.g., heightened stress levels, executive function impairments, dysautonomia, immune dysregulation) may be amenable to chiropractic care, though the evidence base remains nascent. Notably, while a few studies have explored the relationship between chiropractic care and PNI-related outcomes in clinical populations (37, 40, 42), to our knowledge, no such research has been conducted among individuals with obesity.

To bridge this gap in the literature, our laboratory is launching a novel line of research evaluating PNI functioning in adults with obesity undergoing chiropractic care. The primary aim of the current pilot trial was to determine the feasibility of various aspects of our current design (i.e., eligibility, compliance, tolerability, adherence, and retention). The secondary aim was to explore potential changes over time in PNI-related subjective and objective outcomes.

## Methods

### Ethics and trial registration

This trial was approved by Life University’s Institutional Review Board in July 2024 and prospectively registered on ClinicalTrials.gov in October 2024 (NCT06208163). This pilot trial is reported according to the guidance outlined in the Consolidated Standards of Reporting Trials (CONSORT) extension to randomized pilot and feasibility trials (45).

### Design

We conducted a single-arm pilot trial to quantitatively assess key implementation outcomes and determine the feasibility of our design in adults with obesity. While the high prevalence of adult obesity in the US population was a primary factor in this cohort selection, individuals with obesity also frequently exhibit dysregulation across multiple PNI-related domains. As such, adults with obesity represent a relevant clinical population in whom the potential effects of chiropractic care can be meaningfully evaluated in a future definitive trial. Due to resource constraints (i.e., funding and time), a single-arm design was selected for this pilot stage. Importantly, this design lacks a comparative control group and therefore precludes inferring a causal relationship between the chiropractic intervention and changes in secondary PNI outcomes.

### Eligibility criteria

The following eligibility criteria were established for this trial:

- 18–65 years of age.
- BMI ≥30.
- Waist circumference ≥35” for women or ≥40” for men.
- Able to walk unassisted on a treadmill.
- No chiropractic care within 30 days of enrollment.
- Not taking short-acting benzodiazepines (i.e., midazolam; triazolam). If they are taking other prescription medications, they must be on a stable dose for a minimum of 6 weeks with no plans to alter dose(s) during the study.
- No contraindications for rapid postural changes (e.g., postural orthostatic tachycardia syndrome, orthostatic hypotension).
- No heart conditions (including pacemakers) that impact the electrical or mechanical function of the heart.
- Not diagnosed with any externalizing or thought disorders (e.g., substance use disorder, schizophrenia) that are uncontrolled or untreated.
- No hearing impairments.
- No current litigation related to a physical, health-related injury.
- No whiplash injuries within 3 months of enrollment.
- No oral injuries, inflammation, or disease that cause their mouth or gums to bleed easily.
- Not pregnant.

### Trial location

Assessments and interventions took place at the CCR (Marrietta, GA, USA) and participating community chiropractic clinics.

### Trial overview

**Figure 1:**
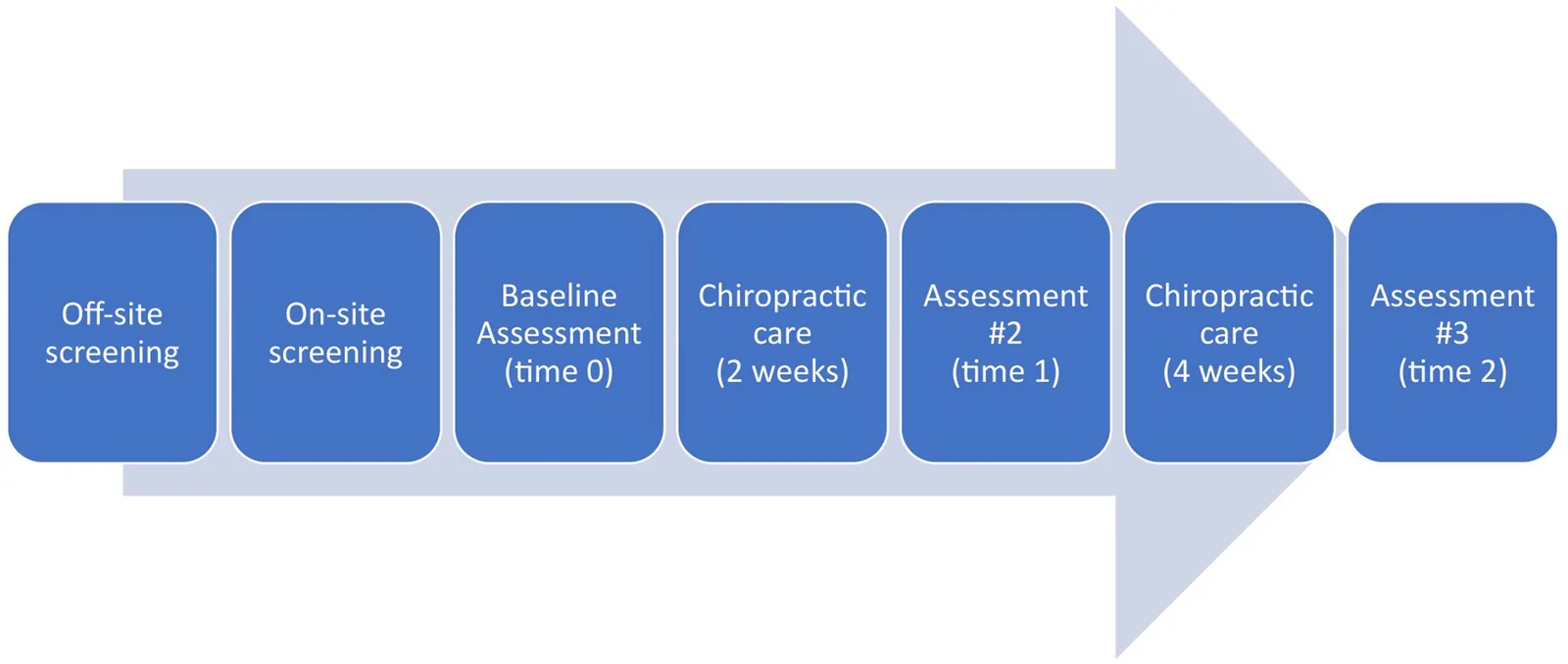
Trial flow.

An overview of the trial flow is shown in **Figure 1**.

#### Off-site screening

Participants were recruited via word of mouth, flyers, social media posts, trial registries, and newspaper advertisements. Recruitment materials contained participation incentives (i.e., complimentary chiropractic care; up to US$205 in gift cards) and a link/QR code to a Health Insurance Portability and Accountability Act (HIPAA) compliant screening survey (JotForm Inc., San Francisco, CA, USA). Individuals who self-reported that they met the eligibility criteria were scheduled for a baseline session at the CCR.

#### On-site screening

Upon arrival at the CCR, anthropometric measurements were collected (i.e., height, weight, waist circumference) to verify eligibility. Waist circumference was determined by wrapping a flexible/soft measuring tape around the abdomen at the level of the umbilicus. Individuals who did not meet eligibility criteria were excluded and compensated with a US$10 gas card. A member of the research staff provided eligible individuals with a detailed description of the trial, advised them of their rights, and asked to sign the written informed consent form. Enrollees were then provided with a quiet room and an electronic tablet to complete a comprehensive intake form querying demographic and health history information.

### Sample size

Following recent guidance for sample size determination in pilot and feasibility studies (46–48), our sample size was based on each primary outcome reaching at least 90% power for rejecting the hypothesis that the outcome falls within the ‘red/stop’ zone if the ‘green/go’ zone holds true. In line with recent guidance, we stated our minimal (min) and hypothesized (hyp) values for each feasibility outcome *a priori*. The minimal values represent the smallest proportion for an outcome that would still allow the possibility of the main study to be successful (with or without amendment). The hypothesized values were those we considered representing feasibility and were as follows: eligibility (min=0.60, hyp=0.90); compliance (min=0.60, hyp=0.90); tolerability (min=0.60, hyp=0.90); adherence (min=0.60, hyp=0.90); and retention (min=0.60, hyp=0.90). This computation was performed using the freely available SS-PROGRESS webapp for sample size calculation and evaluation of progression criteria in pilot and feasibility studies. The required sample size and power for each feasibility objective were as follows: population-level eligibility (n=19; ~90% power), participant-level compliance (n=17; ~92% power), participant-level tolerability (n=17; ~92% power), participant-level adherence (n=17; ~92% power), and participant-level retention (n=17; ~92% power).

### Assessments

Prior to baseline assessment sessions (time 0), an email was sent outlining lifestyle restrictions including abstaining from 1) alcohol, non-prescription medications, and strenuous exercise within 24 hours of the appointment and 2) caffeine, nicotine, food, and rapid ingestion of large quantities of liquids within 3 hours of the appointment. Additionally, reminder texts containing a link to an infographic outlining these restrictions were sent 24 hours and 3 hours prior to every assessment session (timepoints 0, 1, and 2). Adherence to these restrictions was queried prior to initiating assessments. Additionally, to control for the well-documented diurnal variations in neuroimmune markers (49, 50), all assessments for a given individual were performed at approximately the same time of day.

### Saliva collections

**Figure 2:**
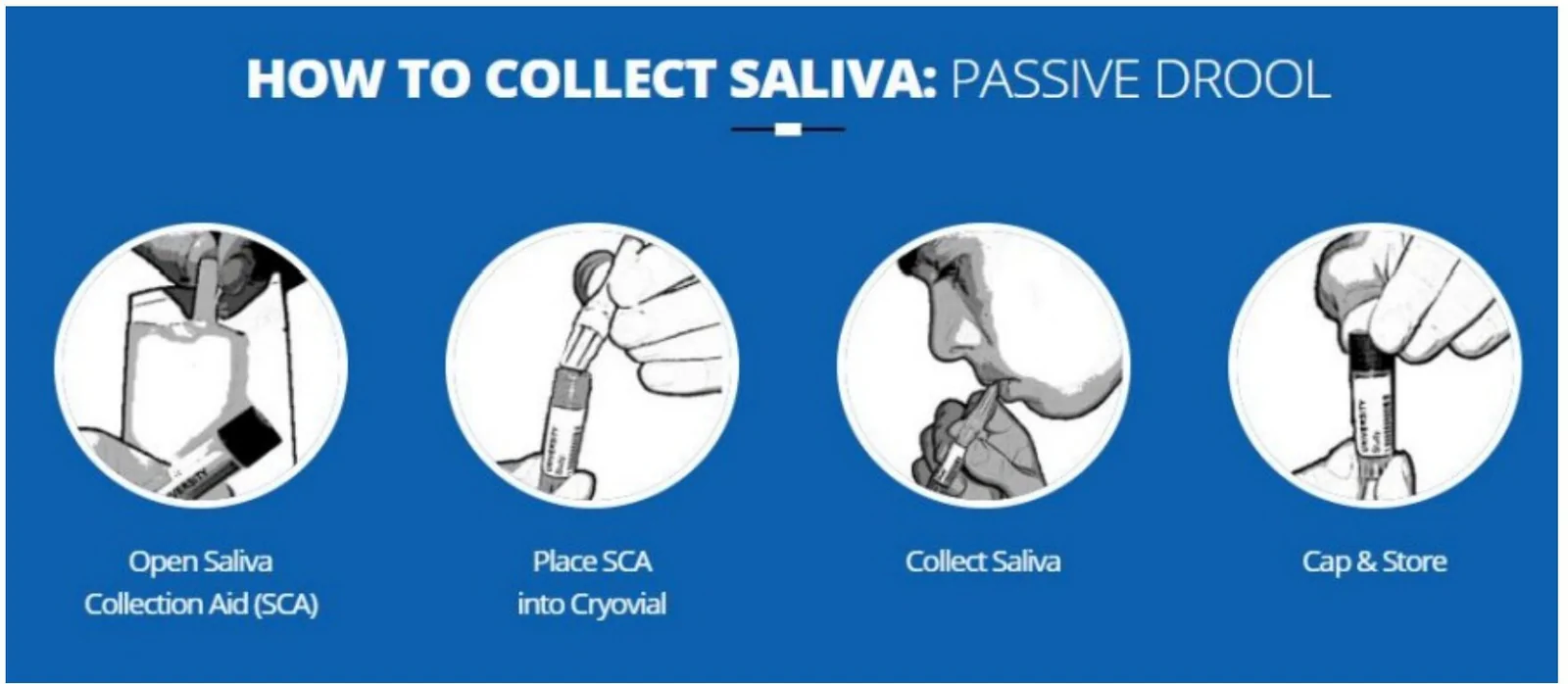
Saliva collection methodology (reproduced with permission from Salimetrics LLC).

Saliva was collected via the passive drool method. Immediately prior to collection, participants were asked to rinse their mouths with water to ensure any food debris were cleared. As illustrated in **Figure 2**, the following methodology was implemented to collect each sample: 1) researcher opened pouch and removed the saliva collection aid, 2) researcher placed ribbed-end of the saliva collection aid securely into a pre-labeled (i.e., alphanumeric designation indicating the participant & sample number) collection vial, 3) researcher gave the vial to the participant, asked the participant to allow saliva to pool in mouth, tilt their head forward, and gently guide saliva through the saliva collection aid into the vial ensuring they do not blow or spit into the vial, 4) researcher capped and immediately stored the specimen in a deep freezer at a temperature of -10°F (-23 °C). Samples were shipped on dry ice and assayed at the Salimetrics SalivaLab (Carlsbad, California, USA) using the Salimetrics Salivary Secretory IgA ELISA Kit (catalog number 1-1602), without modifications to the manufacturer’s protocol.

### Electrocardiography and impedance cardiography acquisitions

**Figure 3:**
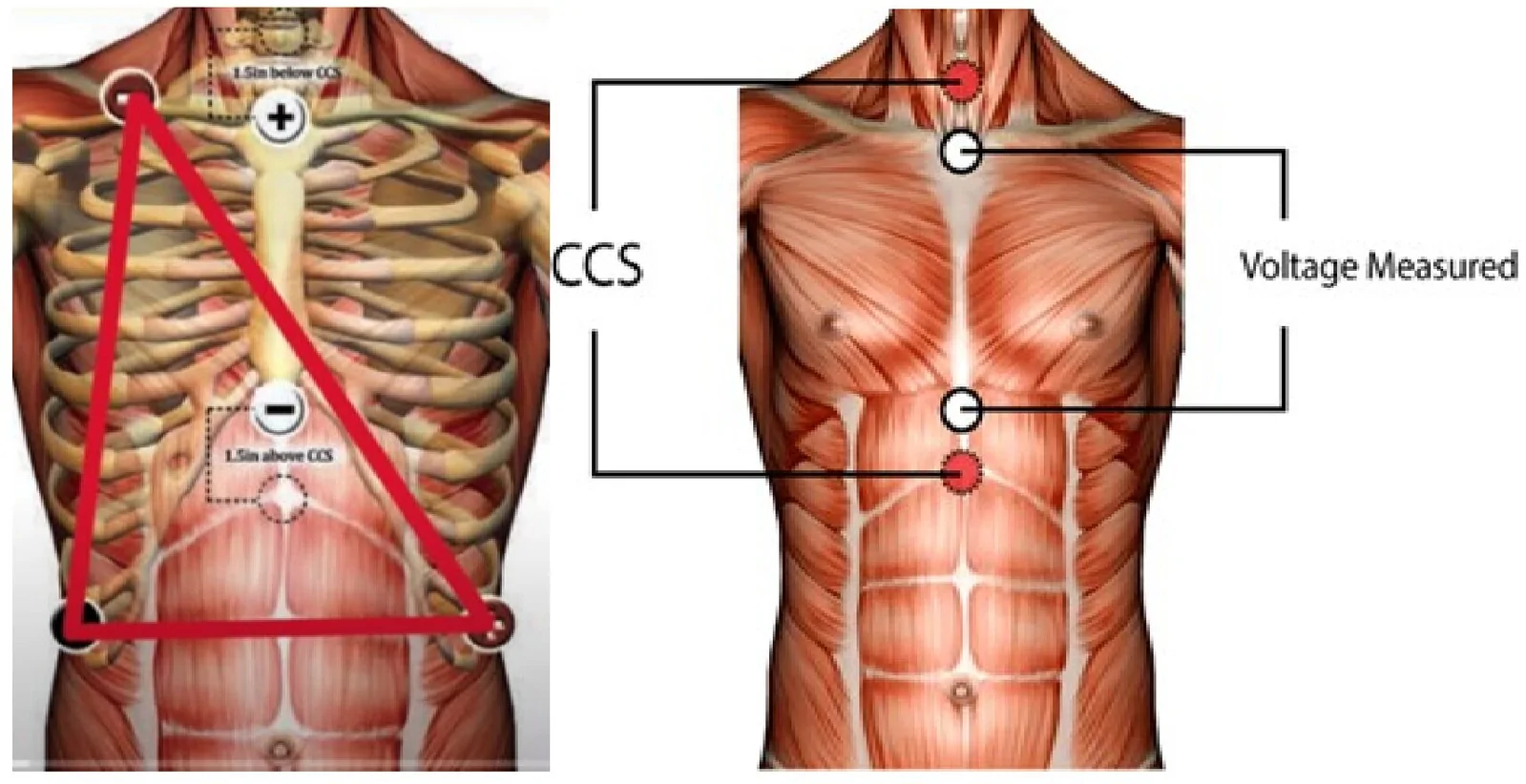
Lead-II ECG (left) and ICG (right) configurations. CCS = constant current source.

Immediately prior to ECG/ICG acquisitions, participants were asked to use the bathroom to empty their bladders. Signals were acquired in a temperature-controlled (~72F) room at a 500 Hz sampling rate using the DC-coupled, 8-channel, Wi-Fi enabled Mindware Mobile amplifier (Mindware Technologies Ltd., Gahanna, OH, USA) and Biolab software (version 3.4.1). All leads were inserted into the Mindware Mobile amplifier and visual checks of each signal were performed to ensure proper polarity and a high signal-to-noise ratio prior to acquisitions. For each acquisition, a researcher prepared each area by rubbing an alcohol wipe in a circular motion until the skin was slightly abraded and red. If necessary, participants were provided with a razor and gel and asked to shave any areas on the thorax where the 1.5” circular foam Ag/AgCl spot electrodes (7% chloride wet gel) were to be applied. To track the heart’s electrical activity, we used a standard lead-II ECG with the negative electrode on the right clavicle, positive electrode at the level of the 10th rib on the participant’s left antero-lateral thorax, and ground electrode attached at the level of the 10th rib on the right antero-lateral thorax (**Figure 3**, left). To track the heart’s mechanical functioning, two ICG sensing electrodes were secured to the anterior thorax with the negative electrode at the xiphoid process and the positive electrode on the sternal notch (**Figure 3**, right). Two ICG constant current source (CCS) electrodes were attached to the posterior thorax with the positive electrode near the inferior aspect of the cervical spine (~4 cm superior to the sternal notch level) and the negative electrode near the inferior aspect of the thoracic spine (~4 cm inferior to the xiphoid level). A continuous, high-frequency (100 kHz), low-amplitude (500 µA) current was passed through the thoracic cavity to quantify impedance levels. The magnitude of the 100 kHz signal received by the sensing electrodes was displayed as the raw impedance waveform (Z_0_). Additionally, the first derivative of Z_0_ (i.e., dZ/dt) was displayed and recorded. A small piece of medical tape was used to create a strain relief loop in each electrode lead wire just proximal to the electrode.

**Figure 4:**
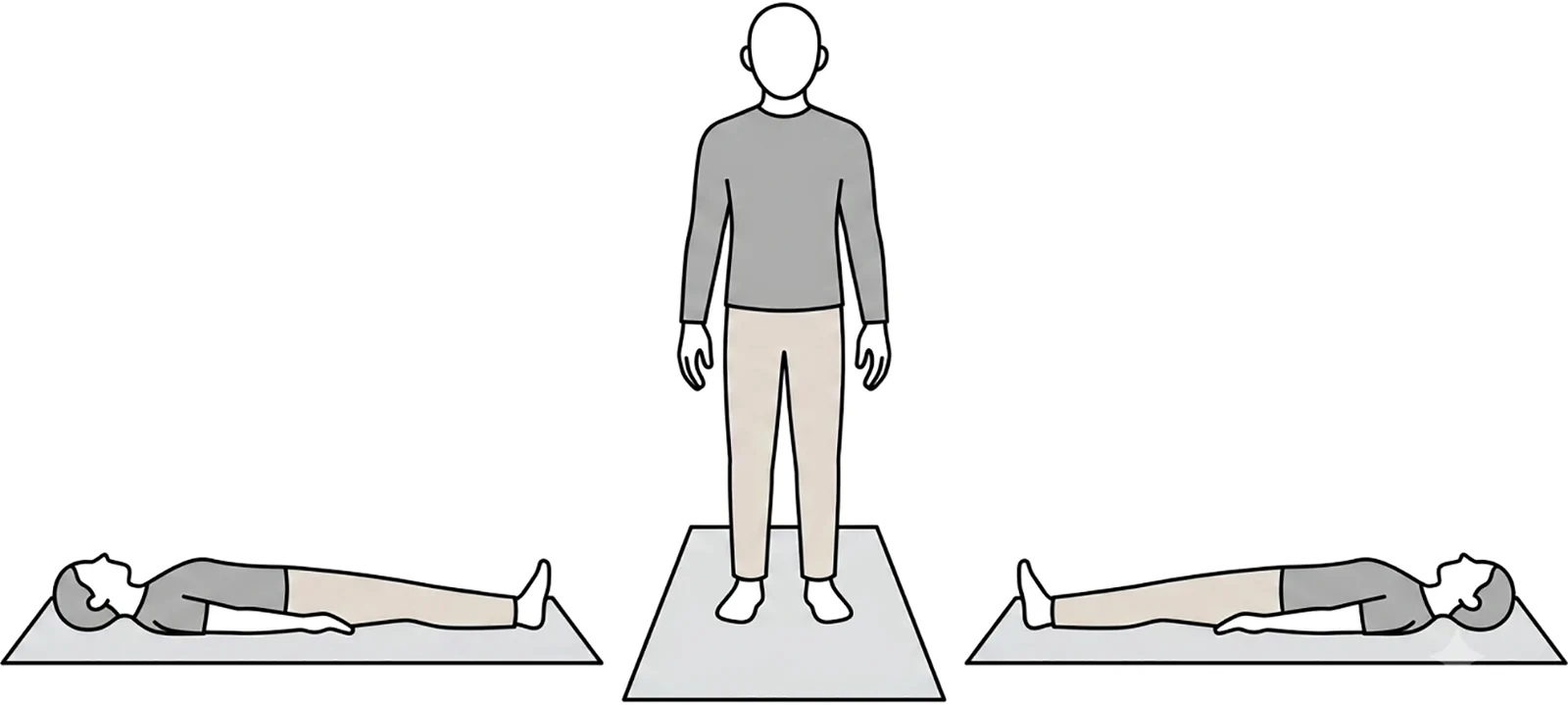
Orthostatic challenge.

As shown in **Figure 4**, participants undertook an orthostatic challenge whereby they were asked to lie supine for a period of ~8 min (5 min acclimation + 3 min resting acquisition), quickly stand and remain standing for ~3 min (reactivity acquisition), and re-assume a supine position for ~3 min (recovery acquisition) while breathing spontaneously. Signals were not visible to the participants during the challenge. This challenge was conducted under the supervision of trained research personnel. Testing was immediately discontinued if contraindications were identified or if participants exhibited symptoms suggestive of autonomic instability (e.g., dizziness, tachycardia, blurred vision).

### Patient-reported outcomes

Research has shown that electronic data collection increases the speed, accuracy, and user acceptability of the process (51–53). As such, all PROs (English versions) were re-created in JotForm and participants were asked to complete the series on an electronic tablet. To prevent missing data, a visual alert was generated if any queries on a given form had missing responses. PROs included the COMPASS-31, PSS-10 (v2.0), PROMIS Cog-8 (v2.0), and PROMIS-29 (v2.1).

The COMPASS-31 is a 31-item questionnaire that evaluates ANS functioning across 6 domains: 1) orthostatic intolerance (4-items), 2) vasomotor (3-items), 3) secretomotor (4-items), 4) gastrointestinal (12-items), 5) bladder (3-items), and 6) pupillomotor (5-items) (20). The domains are weighted, and the sum of the weighted sub-scores yields a total score ranging from 0-100. Total raw scores ≥20 are suggested to reflect moderate-to-severe autonomic dysfunction (54, 55).

The NIH Toolbox PSS-10 is a short (10-item) measure of perceptions of recent stress (11). Total raw scores can range from 10 to 50. NIH Toolbox negative emotion measures utilize a 7-day recall period, 5-point Likert scales (e.g., 1=never, 2=almost never, 3=sometimes, 4=fairly often, 5=very often), and standardized *uncorrected* T-scores (mean=50, SD = 10) using conversion tables available in the online scoring instructions (https://www.healthmeasures.net/). Higher scores indicate greater levels of perceived stress with T-scores ≥60 deemed ‘potentially problematic’.

The PROMIS Cog-8 is a brief (8-item) assessment of subjective cognitive functioning (21). Total raw scores can range from 8 to 40. The PROMIS-29 is a 29-item survey that assesses 8 health domains: 1) physical function, 2) anxiety, 3) depression, 4) fatigue, 5) sleep disturbance, 6) social participation, 7) pain interference, and 8) pain intensity (56). Apart from pain intensity, which is a single item scored on a 1 (no pain) to 10 (worst pain imaginable) scale, total raw scores in each domain can range from 4 to 20. Both PROMIS measures use a 7-day recall period and relevant 5-point Likert scales (e.g., 1=never, 2=rarely, 3=sometimes, 4=often, 5=always). Exempting pain intensity, each (sub)scale score is summed and converted to a standardized T-score (mean=50, SD = 10) using conversion tables available in the scoring instructions at the PROMIS website (https://www.healthmeasures.net/). *Lower* scores on the cognitive function, physical function, and social participation (sub)scales indicate greater functional impairment with standard benchmarks for mild (T-score = 40 to 45), moderate (T-score = 30 to 40), or severe (T-score <30) impairment. In contrast, *higher* scores on the anxiety, depression, fatigue, sleep disturbance, and pain interference subscales indicate greater severity of symptoms with standard benchmarks for mild (T-score = 55 to 60), moderate (T-score = 60 to 70), and severe (T-score >70) symptoms.

### Intervention

The introduction of pragmatic elements into modern trial designs has been increasingly advocated in recent years to provide more “real-world” evidence and enhance the external validity of trial results (57). As such, we chose a pragmatic, hybrid approach which integrated lab-based assessments with community-based chiropractic care. Following baseline assessments, participants were scheduled with a licensed volunteer field clinician practicing in or around Marietta, GA or with our staff clinicians. Participants were permitted to select their clinician based upon the clinic’s proximity to their home or work. Prior to trial initiation, all clinicians were trained on trial protocols, required to complete online ‘Human Subjects Research’ training via the Collaborative Institutional Training Initiative (CITI Program; https://about.citiprogram.org), and were provided with malpractice insurance through Life University. At their initial chiropractic visit, participants underwent a complete physical exam and X-rays (as needed) to ensure there were no contraindications to chiropractic care and determine an appropriate care plan. Consistent with our pragmatic design, vertebral subluxation identification followed each clinician’s routine clinical processes. This approach was selected to reflect real-world practice while maintaining minimal intervention constraints for study consistency. Although protocols varied by clinician, the presence of a vertebral subluxation is generally indicated by a loss of normal intersegmental end feel, palpable restricted intersegmental range of motion in lateral flexion and rotation, and/or tenderness to palpation of the joint (58). Visit frequency and total sessions ranged from 1–3 times per week and 10–14 visits, respectively. Notably, while care plans were permitted to vary based on clinical presentation, clinicians were required to use only high-velocity, low-amplitude (HVLA) thrust techniques to correct subluxations, without incorporating additional modalities (e.g., heat, ice, or electrical stimulation).

### Primary outcomes

As shown in **Table 1**, the primary outcomes included: 1) eligibility, 2) compliance, 3) tolerability, 4) adherence, and 5) retention. Guided by current recommendations for pilot trials (59) we utilized the ‘traffic light’ (red/amber/green; RAG) approach with prespecified progression criteria to determine trial feasibility. Feasibility outcomes and progression criteria were drafted following several rounds of discussions and informed by the existing literature (45, 60, 61) and trialists’ experience. In addition, reasons for dropouts were queried and reported.

**Table 1:** Feasibility outcomes with progression criteria.

Feasibility outcome	Go (green): Feasible without changes	Caution (amber): feasible with minor or major amendments	Stop (red): infeasible
Eligibility Proportion of adults attending on-site screening who are eligible to participate	≥0.90	<0.90 & >0.60	≤0.60
Compliance Proportion of enrollees complying with 24hr and 3hr pre-baseline lifestyle restrictions	≥0.90	<0.90 & >0.60	≤0.60
Tolerability Proportion of enrollees able to complete baseline assessments as directed	≥0.90	<0.90 & >0.60	≤0.60
Adherence Proportion of enrollees attending ≥80% of chiropractic sessions	≥0.90	<0.90 & >0.60	≤0.60
Retention Proportion of enrollees who attend the final assessment session	≥0.90	<0.90 & >0.60	≤0.60

### Secondary outcomes

Secondary outcomes included exploring potential changes over time in 1) HRV and PEP before, during, and after an orthostatic challenge; 2) PROs (PROMIS-29; PROMIS Cog-8; PSS-10; COMPASS-31); and 3) immunological functioning (salivary sIgA levels). Our ECG/ICG pre-processing pipeline can be found in the supplement.

### Statistical considerations

To evaluate primary endpoints, hypothesis testing incorporated α (1-sided) = 5% and power = 90%. The Binomial Exact test was used to compute and plot proportion point estimates in the SS-PROGRESS webapp.

For all secondary endpoints, mixed models were run using custom scripts in R (v4.4.3) (62) utilizing the *mmrm* package (63). Mixed models account for non-independent data (e.g., repeated measures) and have been shown to provide robust estimates in longitudinal designs even with high levels of missing data (64). A Mixed Model for Repeated Measures (MMRM) was implemented within an unconstrained longitudinal framework (65). In this ANCOVA-style approach, post-baseline (2 week and 6 week) values served as the dependent variable, while the baseline score was included as a continuous covariate to account for initial individual differences. This unconstrained formulation was selected for the single-arm design as it treats the observed baseline as a stochastic covariate rather than a fixed constraint. Residuals were modeled using an unstructured covariance matrix (or compound symmetry if convergence was not met) to allow for unique variances and correlations between timepoints. Statistical inference was based on an estimation-focused approach, prioritizing the magnitude of change and the precision of estimates rather than null-hypothesis significance testing. To account for the small sample size, 95% confidence intervals (95% CIs) for all estimates were calculated using the Kenward-Roger degrees of freedom adjustment (66). Model assumptions, including normality of residuals and homoscedasticity, were verified via visual inspection of Q-Q and Residual-vs-Fitted plots. Estimated marginal means for post-baseline timepoints were adjusted for baseline and compared to the observed baseline mean to determine the estimated mean change and associated precision (95% CIs) using the *emmeans* package (67).

## Results

### Recruitment & numbers analyzed

**Figure 5:**
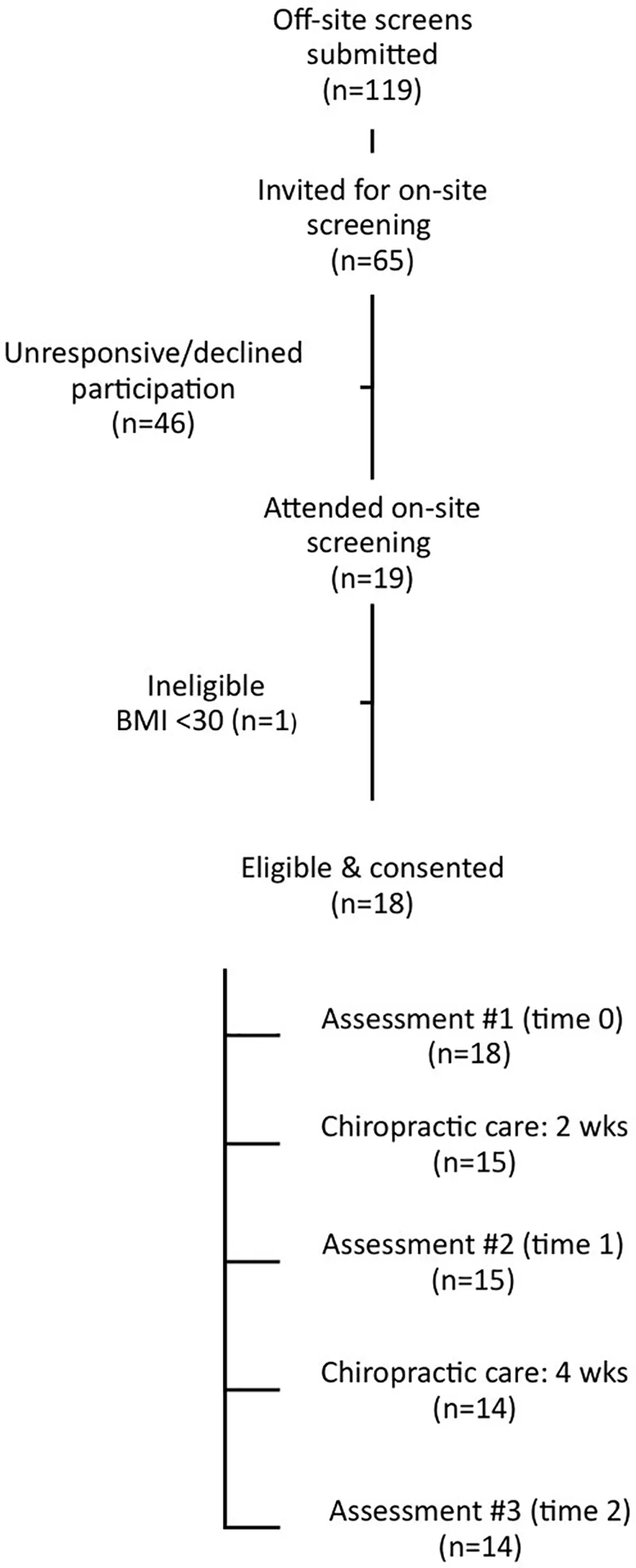
CONSORT flow diagram.

A CONSORT flow diagram is provided (**Figure 5**). Recruitment began on November 11, 2024, and ended on September 4, 2025. Notably, due to time and funding constraints, recruitment ended prior to achieving our target sample size (i.e., n=20). Of those who undertook baseline assessments, 15 attended their initial chiropractic session and were prescribed a chiropractic care plan. Of those who initiated care, 15 and 14 attended assessment sessions 2 and 3, respectively. Reasons for dropout included researcher-initiated withdrawal (n=1; see *tolerability* section below), excessive commute time (n=1), lack of transportation (n=1), and “personal circumstances” (n=1).

### Baseline data

Demographic and baseline clinical characteristics of our entire sample are provided in **Table 2**. Additionally, demographic and baseline clinical characteristics for participants who completed the trial and those that dropped out are shown in **Table 3**. Notably, during the PRO development process, only three of four PROMIS-29 Pain Interference questions were input into JotForm and given to participants; therefore, no data is available for this subscale.

**Table 2:** Demographic and baseline clinical characteristics.

Variables	Participants (n=18)
Age, years*	48.77 (± 9.02)
F:M	12:6
BMI, kg/m^2^*	38.62 (± 6.16)
Waist, inches*	43.44 (± 1.92)
Race	
Black/African American. White/European. Latino/Hispanic. Asian. Mixed.	38.89% (7) 38.89% (7) 5.56% (1) 5.56% (1) 11.11% (2)
PNI-related measures	
COMPASS-31 (raw score)*. PSS-10 (T-score)*. PROMIS Cog-8 (T-score)*. PROMIS-29* Physical Function (T-score). Social Participation (T-score). Anxiety (T-score). Depression (T-score). Fatigue (T-score). Sleep Disturbance (T-score). Pain Intensity (raw score). Pain Interference (T-score). RMSSD (msec)**. resting. reactivity. recovery. PEP (msec)** resting. reactivity. recovery. Salivary sIgA (µg/mL)**.	35.35 (± 10.55) 51.69 (± 7.79) 50.97 (± 6.66) 51.35 (± 6.37) 55.54 (± 7.97) 50.27 (± 7.67) 47.39 (± 8.47) 50.78 (± 8.77) 49.06 (± 6.21) 3.12 (± 1.65) no data 24.0 [16.2, 32.1] 13.6 [8.6, 16.7] 25.3 [21.4, 35.6] 89.5 [71.8, 101.5] 61.0 [51.0, 77.0] 93.0 [74.0, 96.8] 216.4 [131.7, 389.6]

*mean (standard deviation); **median [IQR]; F:M, female to male ratio; msec, milliseconds; µg/mL, micrograms per milliliter.

**Table 3:** Demographic and baseline clinical characteristics for trial completers and dropouts.

Variables	Completers (n=14)	Dropouts (n=4)
Age, years*	47.86 (± 9.53)	51.50 (± 7.33)
F:M	8:6	4:0
BMI, kg/m^2^*	39.81 (± 5.97)	34.42 (± 5.51)
Waist, inches*	43.93 (± 1.86)	41.75 (± 0.96)
Race	
Black/African American. White/European. Latino/Hispanic. Asian. Mixed.	42.86% (6) 28.57% (4) 14.29% (2) 7.14% (1) 7.14% (1)	25.00% (1) 50.00% (2) 25.00% (1) 0.00% (0) 0.00% (0)
PNI-related measures	
COMPASS-31 (raw score)*. PSS-10 (T-score)*. PROMIS Cog-8 (T-score)*. PROMIS-29* Physical Function (T-score). Social Participation (T-score). Anxiety (T-score). Depression (T-score). Fatigue (T-score). Sleep Disturbance (T-score). Pain Intensity (raw score). Pain Interference (T-score). RMSSD (msec)**. resting. reactivity. recovery. PEP (msec)** resting. reactivity. recovery. Salivary sIgA (µg/mL)**.	36.00 (± 11.92) 50.18 (± 6.98) 50.10 (± 6.97) 51.66 (± 6.19) 55.80 (± 8.24) 50.22 (± 7.91) 45.74 (± 8.00) 51.02 (± 7.84) 49.18 (± 6.72) 3.38 (± 1.80) No data 26.9 [15.5, 33.1] 12.8 [7.9, 20.5] 29.0 [21.9, 36.1] 90.0 [89.0, 103.0] 62.0 [50.8, 77.2] 94.0 [86.2, 96.9] 171.9 [129.8, 379.9]	33.25 (± 4.27) 56.60 (± 9.34) 53.78 (± 5.31) 50.35 (± 7.82) 54.67 (± 8.11) 50.45 (± 7.96) 52.75 (± 8.76) 50.00 (± 12.79) 48.67 (± 4.95) 2.25 (± 0.50) No data 21.2 [21.2, 21.2] 16.7 [16.7, 16.7] 16.4 [16.4, 16.4] 65.0 [65.0, 65.0] 61.0 [61.0, 61.0] 54.0 [54.0, 54.0] 306.2 [221.7, 715.3]

*mean (standard deviation); **median [IQR]; F:M, female to male ratio; msec, milliseconds; µg/mL, micrograms per milliliter. Due to technical equipment errors during the baseline physiological laboratory sessions, ECG and impedance cardiography data were unrecoverable for 3 participants, resulting in n=1 for baseline RMSSD and PEP metrics within the dropout cohort.

### Primary outcomes

**Figure 6:**
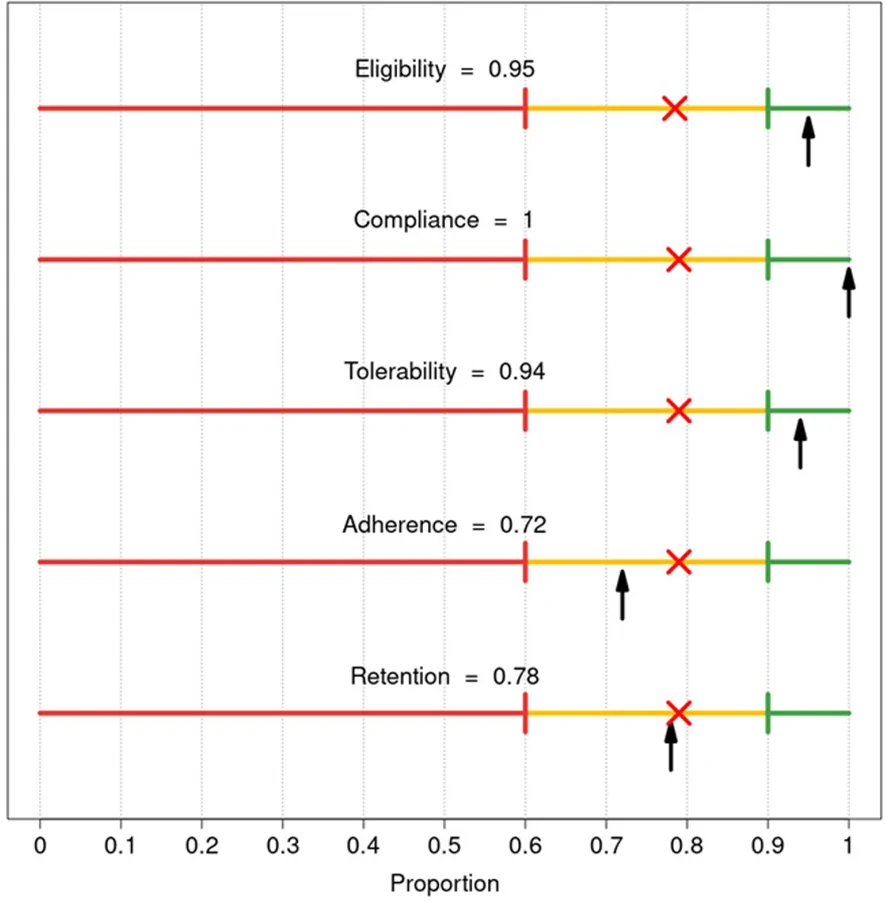
Feasibility outcome results. green region = go/feasible without amendments; yellow/amber region = caution/feasible with minor or major amendments; red region = stop/infeasible; black arrows = proportion point estimates; red cross = critical value for rejecting the null.

A summary of the results is shown in **Figure 6**.

### Eligibility

The point estimate for the proportion of adults eligible following the on-site screening was 0.95 (i.e., 18 of 19). This falls within the ‘green’ and above the critical value for rejecting the null, indicating that this aspect of the trial is feasible without amendments.

### Compliance

The point estimate for the proportion of enrollees complying with both 24-hour and 3-hour lifestyle restrictions was 1.00 (18 of 18). This falls within the ‘green’ zone and above the critical value for rejecting the null, indicating that this aspect of the trial is feasible without amendments.

### Tolerability

The point estimate for the proportion of enrollees able to complete all baseline assessments as directed was 0.94 (17 of 18). This falls within the prespecified ‘green’ zone and above the critical value for rejecting the null, indicating that this aspect of the trial is feasible without amendments. Notably, one participant was withdrawn by the research staff prior to the orthostatic challenge after they disclosed a diagnosis of postural orthostatic tachycardia syndrome (POTS). This condition represents a contraindication to the challenge given its potential to provoke an adverse autonomic response.

### Adherence

The point estimate for the proportion of enrollees attending ≥80% of chiropractic sessions was 0.72 (13 of 18). This falls within the ‘yellow/amber’ zone and is below the critical value for rejecting the null hypothesis, indicating that this aspect of the trial is feasible only with major amendments. Of the five non-adherent participants, three dropped out prior to their first session, while the remaining two attended ~77% and ~12% of their scheduled appointments, respectively.

### Retention

The point estimate for the proportion of enrollees retained in the trial was 0.78 (14 of 18). This falls within the ‘yellow’ zone and just below the critical value for rejecting the null, indicating that this aspect of the trial is feasible with major amendments. As stated above, reasons for dropout included researcher-initiated withdrawal (n=1), excessive commute time (n=1), lack of transportation (n=1), and “personal circumstances” (n=1).

### Secondary outcomes

### PROs

**Table 4** shows the change scores at each timepoint for each PRO (sub)scale. Plots for all PRO (sub)scales can be found in the supplement (**Supplementary Figures S1–S10**).

**Table 4:** Model-adjusted change from baseline in PRO (sub)scales [95% CIs].

PRO (sub)scale	2-week Δ (timepoint 1)	6-week Δ (timepoint 2)
COMPASS-31*	-5.23 [-6.56 to -3.90]	-4.28 [-6.33 to -2.23]
PSS-10**	-6.82 [-9.86 to -3.78]	-4.93 [-8.36 to -1.50]
PROMIS-Cog 8**	3.13 [0.20 to 6.05]	1.59 [-0.22 to 3.40]
PROMIS-29 Physical Function**	1.52 [-1.51 to 4.55]	2.81 [0.24 to 5.37]
PROMIS-29 Social Participation**	-0.77 [-3.36 to 1.83]	3.12 [0.24 to 6.00]
PROMIS-29 Anxiety**	-2.46 [-6.15 to 1.23]	-2.87 [-7.45 to 1.71]
PROMIS-29 Depression**	-3.29 [-4.35 to -2.24]	-1.69 [-4.34 to 0.97]
PROMIS-29 Fatigue**	-1.22 [-5.10 to 2.65]	-2.35 [-7.10 to 2.39]
PROMIS-29 Sleep Disturbance**	-1.21 [-3.40 to 0.98]	-4.14 [-7.80 to -0.49]
PROMIS-29 Pain Intensity*	-0.36 [-0.98 to 0.26]	0.49 [-0.15 to 1.14]

**Δ**, change; *raw score; **T-score.

### RMSSD

**Figure 7:**
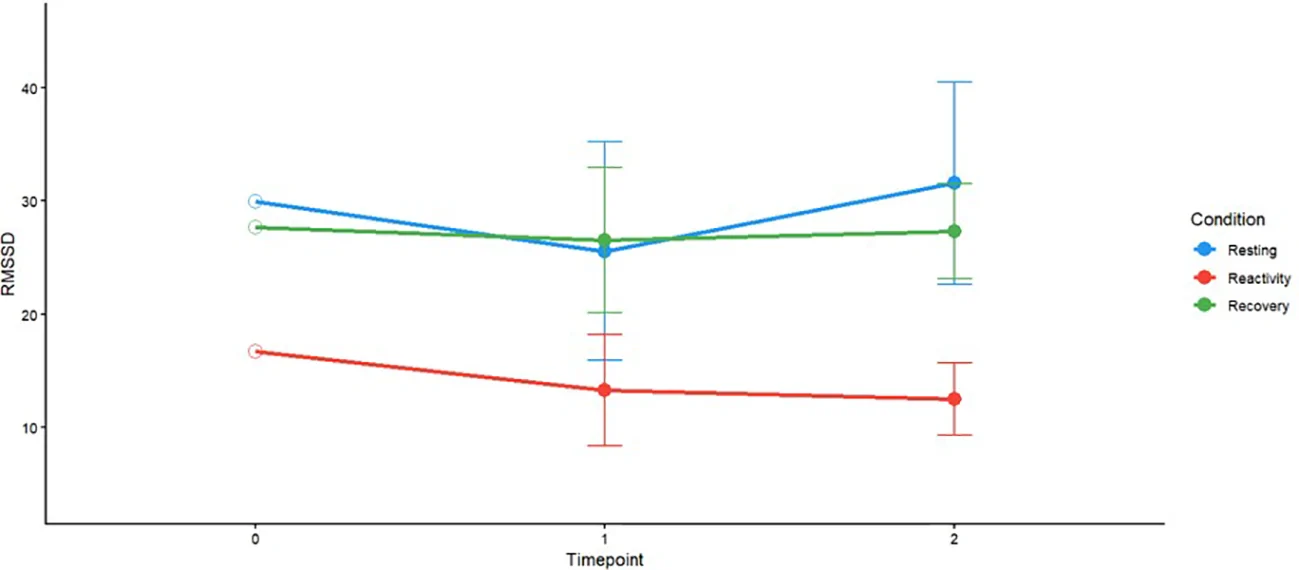
RMSSD (msec) at each timepoint for each condition. Open circles = observed raw mean (timepoint 0); Filled circles = model-adjusted estimates (timepoints 1 and 2); vertical bars = 95% CIs.

**Figure 7** shows RMSSD (msec) values at each timepoint for each condition. In the resting condition, change from baseline at timepoints 1 and 2 were -4.36 (95% CI, -14.04 to 5.32) and 1.64 (95% CI, -7.28 to 10.55), respectively. In the reactivity condition, change from baseline at timepoints 1 and 2 were -3.39 (95% CI, -8.34 to 1.56) and -4.19 (95% CI, -7.41 to -0.97), respectively. In the recovery condition, change from baseline at timepoints 1 and 2 were -1.12 (95% CI, -7.54 to 5.29) and -0.36 (95% CI, -4.55 to 3.83), respectively.

### PEP

**Figure 8:**
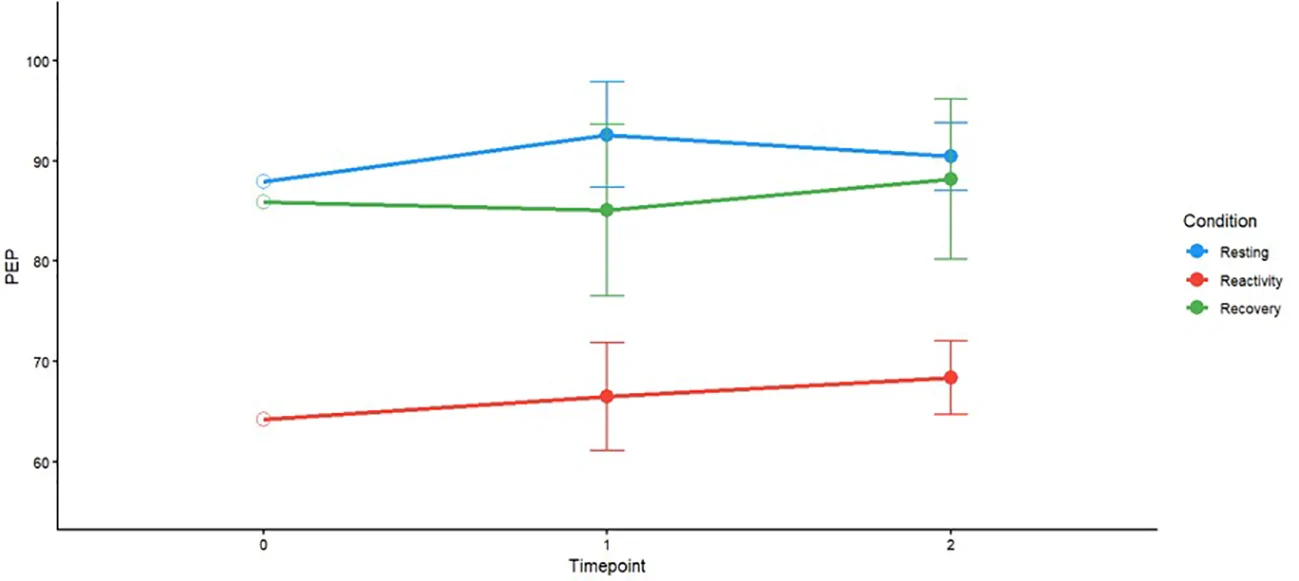
PEP (msec) at each timepoint for each condition. Open circles = observed raw mean (timepoint 0); Filled circles = model-adjusted estimates (timepoints 1 and 2); vertical bars = 95% CIs.

**Figure 8** shows PEP (msec) values at each timepoint for each condition. In the resting condition, change from baseline at timepoints 1 and 2 were 4.71 (95% CI, -0.52 to 9.95) and 2.55 (95% CI, -0.84 to 5.94), respectively. In the reactivity condition, change from baseline at timepoints 1 and 2 were 2.26 (95% CI, -3.11 to 7.63) and 4.14 (95% CI, 0.46 to 7.81), respectively. In the recovery condition, change from baseline at timepoints 1 and 2 were -0.80 (95% CI, -9.35 to 7.75) and 2.32 (95% CI, -5.68 to 10.32), respectively.

### Salivary sIgA

**Figure 9:**
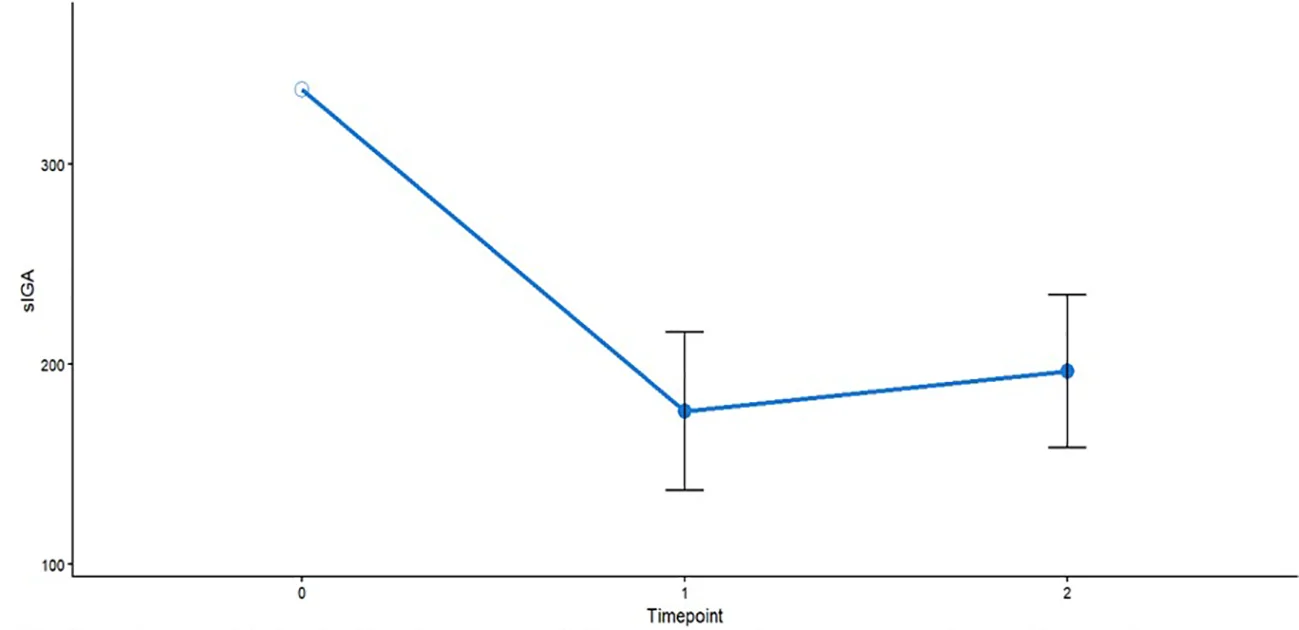
Salivary sIgA (µg/mL) values at each timepoint. Open circles = observed raw mean (timepoint 0); Filled circles = model-adjusted estimates (timepoints 1 and 2); vertical bars = 95% CIs.

**Figure 9** shows salivary sIgA (µg/mL) values across time. Change from baseline at timepoints 1 and 2 were -160.59 (95% CI, -200.21 to -120.98) and -140.49 (95% CI, -178.67 to -102.31), respectively.

## Discussion

The primary aim of this pilot trial was to evaluate various aspects of feasibility when employing a PNI-focused assessment battery in tandem with a community-based chiropractic intervention in an obese adult population. Secondarily, we set out to explore potential changes in PNI-related outcomes over time.

Encouragingly, all primary endpoints indicated that our trial design is feasible. More specifically, point estimates for ‘eligibility’, ‘compliance’, and ‘tolerability’ fell within the green zone (i.e., feasible without amendments). Even when considering the 95% CI lower bounds, these aspects of our design appear to be feasible, albeit with minor amendments. Regarding ‘eligibility’, results suggest that our online screening procedures are robust at filtering out non-eligible individuals. Further, the imposed lifestyle restrictions and assessment battery appeared to be very well accepted and tolerated. Importantly, lifestyle restrictions were implemented based on current guidance for neurophysiological data collection (68–70) designed to mitigate modulation of neurophysiological output due to nuisance factors such as the degree of gastric (70, 71) and bladder distention (69, 72), and circulating levels of caffeine (73, 74), nicotine (75, 76), and alcohol (77, 78). It should be noted that, while compliance with the lifestyle restrictions was reported to be 100%, this metric relied entirely on participant self-report without objective verification. Consequently, this introduces potential social desirability and/or recall biases, representing a limitation that could affect the validity of the results.

With respect to ‘adherence’ and ‘retention’, point estimates suggest that our design is feasible with major amendments. It is plausible that the logistical challenges associated with the hybrid model negatively impacted both adherence and retention in the current trial. Specifically, coordination difficulties between community clinics and lab staff may have contributed to scheduling delays or missed reassessments, while participant-reported inconvenience of attending two separate locations may have increased dropout risk or reduced engagement with the care protocol. If so, the feasibility metrics observed here may represent a conservative estimate of what could be achieved under a more streamlined design, and future trials should consider whether a fully lab-based or fully practice-based model would yield more favorable feasibility outcomes.

Turning our attention to our secondary endpoints, it should be re-iterated that this trial was not powered to assess efficacy outcomes and lacked a comparator control group; therefore, results must be interpreted with considerable caution. In the absence of a control arm, observed within-group changes cannot be attributed to the chiropractic intervention, as spontaneous improvement, regression to the mean, and natural fluctuation in symptom burden represent plausible alternative explanations. With this important caveat in mind, autonomic functioning (i.e., COMPASS-31) and perceived stress levels (i.e., PSS-10) demonstrated the largest within-group changes. This contrasts somewhat with the PROMIS (sub)scales which showed very modest changes over time. Although speculative, this may be partially explained by examining baseline scores. Specifically, the COMPASS-31 mean baseline score was >35, a level which has been suggested to reflect moderate-to-severe dysautonomia (54, 55). In contrast, all PROMIS (sub)scale mean scores fell within ‘normal limits’ (i.e., within ±1 SD of the U.S. population mean) suggesting relatively healthy functioning pre-trial. That said, this doesn’t explain the marked reduction in the mean PSS-10 score which also had a baseline mean within normal limits. Whatever the cause, these self-reported changes are encouraging considering the increasing recognition by patients, clinicians, and regulators of the value of moving towards patient-centered outcomes research which utilizes clinically relevant outcomes that directly capture patients’ perspectives and experiences (79, 80). Definitive trials adequately powered to detect changes in clinical endpoints are required to (dis)confirm these findings.

With respect to objective outcomes, there was little evidence for changes in ANS-related (i.e., RMSSD and PEP) activity in any condition (i.e., resting, reactivity, recovery) over the 6-week course of care. This may reflect relatively preserved cardiac autonomic function at baseline in our sample. Resting RMSSD in our sample (median = 24 msec, IQR: 16–32 msec) was somewhat lower than the reference median reported by Nunan et al. (81) for healthy adults (median = 37 msec, range = 19–75 msec), though values remained within the normative range. To our knowledge, no established population norms exist for short-term PEP. The discrepancy between within-group shifts in ANS-related objective and subjective outcomes is notable and may reflect several non-mutually exclusive explanations. First, the outcomes differ in clinical scope: the COMPASS-31 assesses ANS functioning across multiple end-organs (e.g., eyes, cardiovascular system, gastrointestinal tract, skin, urinary bladder), whereas HRV and PEP index autonomic modulation at the cardiac level and may not capture meaningful changes in other physiological systems. Second, the measures differ in temporal sensitivity: the COMPASS-31 reflects symptom burden integrated over time, while HRV and PEP represent discrete, momentary physiological states that may not capture episodic or cumulative ANS changes. Together, these differences suggest that the two outcome types are complementary rather than redundant, and their divergence may be informative regarding the breadth of ANS involvement in this population. Future work incorporating multi-organ objective ANS assessments (e.g., cardiac, sudomotor, and pupillometric indices) would help clarify this discrepancy.

Interestingly, there was a persistent reduction in salivary sIgA levels, with only a negligible attenuation in the magnitude of change from baseline between timepoints 1 and 2. This contrasts with a recent observational study which followed forty-one participants receiving a single upper cervical chiropractic adjustment, and showed an increase in sIgA levels immediately and at 2 weeks follow-up (44). However, drawing firm comparisons between these trials is tenuous given major differences in design, population, and follow-up duration, and neither was adequately powered for this outcome. From a physiological standpoint, several factors may help contextualize the observed pattern. While acute stressors have been shown to transiently increase sIgA via sympathetic nervous system activation, chronic or repeated stress exposure is associated with sustained reductions in sIgA, likely mediated by prolonged glucocorticoid release and HPA axis activation (82, 83). The repeated procedural demands of ongoing chiropractic care across the study period may have functioned as a source of repeated mild physiological stress, providing one possible explanation for the sustained sIgA reductions observed in our trial. Importantly, saliva samples were collected at the same time of day for each participant across all assessment points, minimizing the potential confounding influence of diurnal variation; a notable methodological strength given that sIgA concentrations are known to fluctuate substantially across the day. Whether sIgA levels would normalize following cessation of care remains an open question that future trials with longer follow-up could address.

The first key limitation of our pilot study was the lack of a control arm. This limitation is important for two reasons. First and foremost, certain feasibility outcomes may be impacted by the presence of a control group. For example, adherence and retention rates may be very different for controls who are not receiving chiropractic care. Second, without a comparison group, interpretation of results is complicated as it is not possible to confidently attribute efficacy to the intervention. For example, changes observed in single-arm trials may be due to the natural course of the condition and/or non-specific (e.g., placebo) effects (84). To address these shortcomings, future trials will consider incorporating a randomized controlled design. Second, due to the small sample size, there was considerable uncertainty in our preliminary estimates of efficacy as evidenced by relatively wide 95% CIs. As this pilot trial was powered for feasibility outcomes rather than efficacy, definitive trials adequately powered for efficacy are required to (dis)confirm these preliminary findings and provide stable effect size estimates. Third, the sex composition of our sample warrants consideration when interpreting results. Females comprised 67% of enrolled participants (12 of 18), which may limit the generalizability of findings across sexes. Importantly, several outcomes assessed in this trial are known to exhibit sex-based differences in the literature. Specifically, HRV indices such as RMSSD tend to be higher in premenopausal females compared to age-matched males, though this difference attenuates with age and is influenced by menopausal status (85, 86). Similarly, perceived stress as measured by the PSS-10 has been shown to differ between sexes, with females generally reporting higher stress levels (87, 88). The predominance of females in the current sample may therefore have influenced the observed baseline values and within-group change estimates for these outcomes. Future definitive trials should aim for a more balanced sex distribution or incorporate sex as a covariate in the analytical model to account for these potential confounding effects. Finally, a notable protocol deviation occurred regarding the PROMIS-29 instrument, where data for the Pain Interference subscale were completely unavailable due to an initial electronic database programming error. This missing data limits the comprehensive characterization of this clinical domain within our cohort. To prevent similar occurrences in future feasibility trials, trialists are strongly encouraged to implement rigorous, multi-user internal pilot testing of all electronic PRO capture systems prior to formal trial initiation.

## Conclusion

Concerning data from across the globe reveals a continuing rise in the rates of obesity, a condition known to be associated with PNI dysfunction. Chiropractic care has been theorized to impact PNI functioning; however, trials substantiating this theory are lacking in most clinical populations. As an important first step, we set out to pilot a novel PNI-related assessment battery in combination with community-based chiropractic care on an obese adult population. Our data suggests that our protocols and procedures are feasible with some revisions. Further, among the PNI-related outcomes assessed in our sample, self-reported autonomic functioning and stress levels showed the most within-group shifts during the trial. Informed by the results from this trial, we are planning a future definitive controlled trial aimed at assessing the efficacy of chiropractic care on PNI-related outcomes in adults with obesity.

## Data Availability

Deidentified individual participant data (IPD) and analytic code have been made publicly available in a data repository (https://doi.org/10.6084/m9.figshare.c.8365459). IPD and analytic code are openly accessible under a Creative Commons Attribution 4.0 International License (CC BY 4.0).
