# Supplementary material for: Psychoneuroimmunological changes in adults with obesity receiving chiropractic care: a single-arm pilot trial
Source: Front Psychiatry. 2026 Jul 17;17:1846987. doi: 10.3389/fpsyt.2026.1846987 (PMC13426054; doi:10.3389/fpsyt.2026.1846987)
Supplement: Supplementary file 1 [file DataSheet1.docx]

## Supplement:

#### ECG/ICG preprocessing

ECG/ICG signal pre-processing was performed offline using freely available Biolab software (version 3.4.1; <https://support.mindwaretech.com/downloads/>) and Autonomic Nervous System Laboratory (ANSLAB) Professional software (version 2.6; <https://www.anslab.net>) running on MATLAB 2022a (MathWorks Inc., Natick, MA, USA.). ANSLAB is a proprietary software suite that permits data visualization, artifact detection, data reduction, and statistical analysis for a wide range of ANS-related signals via a graphical user interface (GUI) (1). Raw Mindware (.mwi) files were individually loaded into Biolab. Excluding postural transition periods, the initial 3-min of the data from each condition (i.e., resting, reactivity, recovery) was isolated, and the files exported in ASCII (.txt) format into a trial folder created within ANSLAB. Per ANSLAB’s conventions, trial folders were named using a 3-letter study identifier (i.e., OBE), and ASCII files were named using the 3-letter study identifier, 3-digit participant identifier, and 2-digit session number (e.g., obe00101). Each ASCII file contained the sampling rate in row 1, tab-delimited channel names in row 2, and tab-delimited data for each channel in subsequent rows.

Each raw ECG time-series was bandpass filtered (0.5-40 Hz) and loaded into the ECG module. ANSLAB’s algorithm automatically detects and labels R-peaks. The ECG signal was visually inspected and, when necessary, manually labeled (if an R-peak was missed by the algorithm) and/or de-artifacted (if noise was misidentified as an R-peak or a non-sinus R-peak from an ectopic beat was labelled). Linear interpolation was employed where necessary. Any data files with >5% interpolated R-peaks were flagged and deemed ineligible for subsequent HRV analysis. After editing, the interbeat interval (IBI) time-series was saved in the study’s ECG folder in MATLAB (.m) format at 4 Hz resolution achieved via weighted average interpolation (2). This reduced IBI time-series was then loaded into ANSLAB’s ‘spectral module’ for analysis. Via ANSLAB’s GUI, MATLAB timing files were manually created within the study’s “raw” folder for each ASCII file which segmented the signal into 60-sec non-overlapping epochs. The root mean square of successive differences (RMSSD) for each segment was automatically derived by ANSLAB. Via a custom script developed in MATLAB, segment-wise RMSSD values (msec) were extracted and exported in Excel (.xlsx) format. The median segment value for each subject was computed and used in the group-level analysis. RMSSD is time-domain HRV metric generally considered a reliable indicator of cardiac-related PSNS activity (3).

Each ICG time-series was high pass (1 Hz) and notch (60 Hz) filtered and loaded along with its associated timing file into the ICG module. To permit segment-wise analysis, timing files were manually created within the study’s “raw” folder for each ASCII file which divided the ICG signal into 60-sec, non-overlapping epochs. Synchronized by the peak of the ECG Q-point (beat-by-beat detection, backward search window = 100 msec, ratio of slope = 0.2), median ensemble averaging of dZ/dt waveforms) was performed on all 60-sec segments meeting the minimum threshold of 15 valid beats. Per ANSLAB’s default parameters (1), waveforms > +/- 2 standard deviations from the mean in the B-point window were considered outliers and automatically excluded. For each ensemble, ANSLAB labeled the B-point, Z-point, and X-point along with their standard error margins within their respective search windows. The B-point detection algorithm was set to ‘notch’ which defaults to ‘zero-crossing’ if ANSLAB is unable to detect a valid B-point. Notably, we ensured that the same algorithm was applied across all valid segments for a given participant. Due to inter-individual waveform variability (i.e., fidelity and depth of the B-wave), the B-point detection sensitivity and search window positions were manually adjusted for each participant to optimize detection. Each ensemble-averaged waveform was visually inspected. Waveforms without valid B-points were rejected. Once the B-point for all valid ensembles has been established and accepted, the PEP (msec) for each segment was automatically derived by ANSLAB. Via a custom script developed in MATLAB, segment-wise PEP values for each subject were extracted and exported in Excel (.xlsx) format. The median segment PEP value for each subject was then computed and used in the group-level analysis. The duration between ECG-derived electrical activity (i.e., onset of left ventricular depolarization = Q-wave) and ICG-derived mechanical activity (i.e., opening of the aortic value = B-point on the dZ/dt trace) is termed the PEP which is considered a valid metric for estimating the degree of cardiac-related SNS activity (4–6).

1. Blechert J, Peyk P, Liedlgruber M, Wilhelm FH. ANSLAB: Integrated multichannel peripheral biosignal processing in psychophysiological science. Behav Res Methods. 2016 Dec;48(4):1528–45. doi:10.3758/s13428-015-0665-1 PubMed PMID: 26511369.

2. Cheung MN, Porges SW. Respiratory influences on cardiac responses during attention. Physiological Psychology. 1977;5(1):53–7.

3. Laborde S, Mosley E, Thayer JF. Heart Rate Variability and Cardiac Vagal Tone in Psychophysiological Research - Recommendations for Experiment Planning, Data Analysis, and Data Reporting. Front Psychol. 2017;8:213. doi:10.3389/fpsyg.2017.00213 PubMed PMID: 28265249; PubMed Central PMCID: PMC5316555.

4. Mansouri S, Alhadidi T, Chabchoub S, Salah RB. Impedance cardiography: recent applications and developments. Biomedical Research. 2018;29(19):3542–52.

5. Krohova J, Czippelova B, Turianikova Z, Lazarova Z, Tonhajzerova I, Javorka M. Preejection period as a sympathetic activity index: a role of confounding factors. Physiol Res. 2017 Sep 22;66(Suppl 2):S265–75. doi:10.33549/physiolres.933682 PubMed PMID: 28937241.

6. Kelsey RM. Beta-adrenergic cardiovascular reactivity and adaptation to stress: The cardiac pre-ejection period as an index of effort. 2012.

### PRO (sub)scale Mixed Model Plots


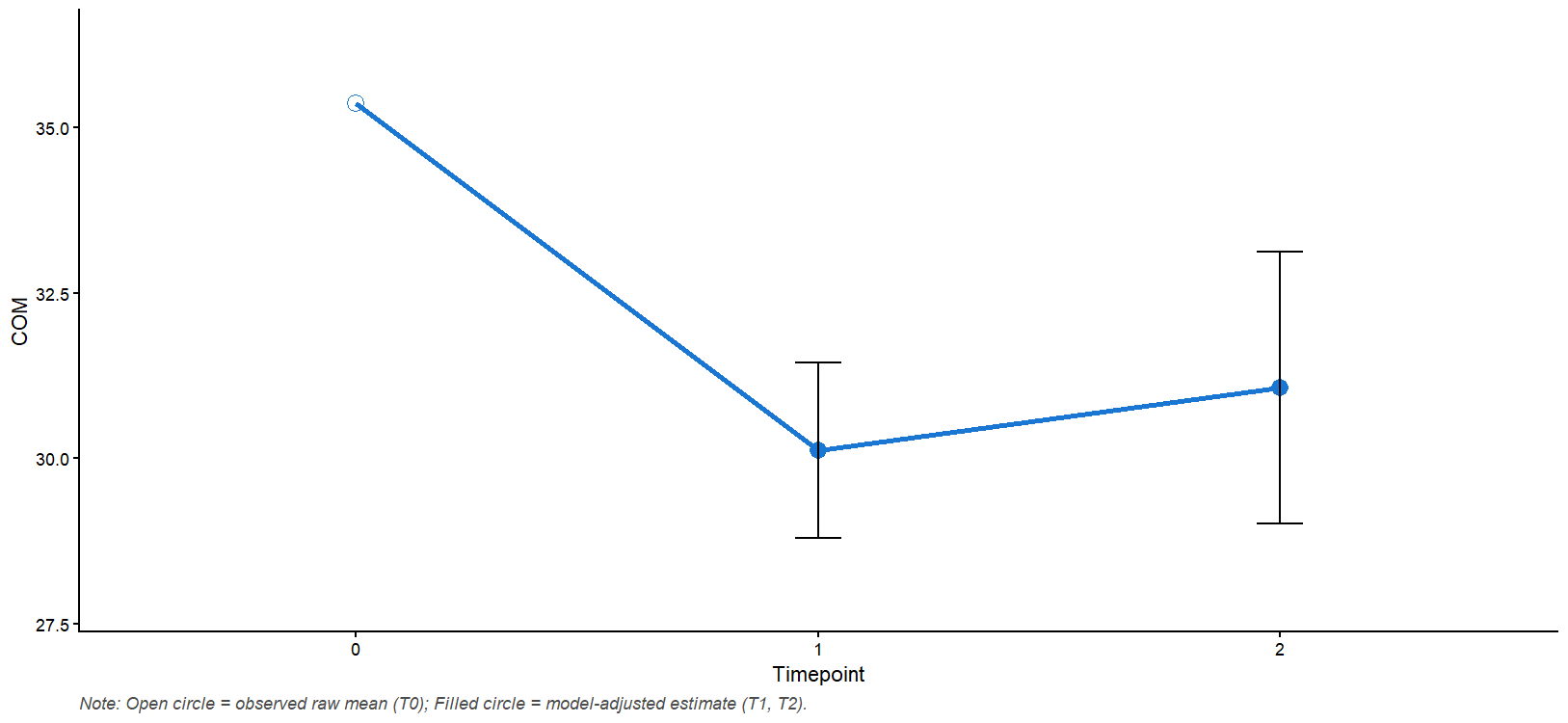


**Fig. S1**: COMPASS-31 scores at each time point. Note: vertical bars = 95% CIs.


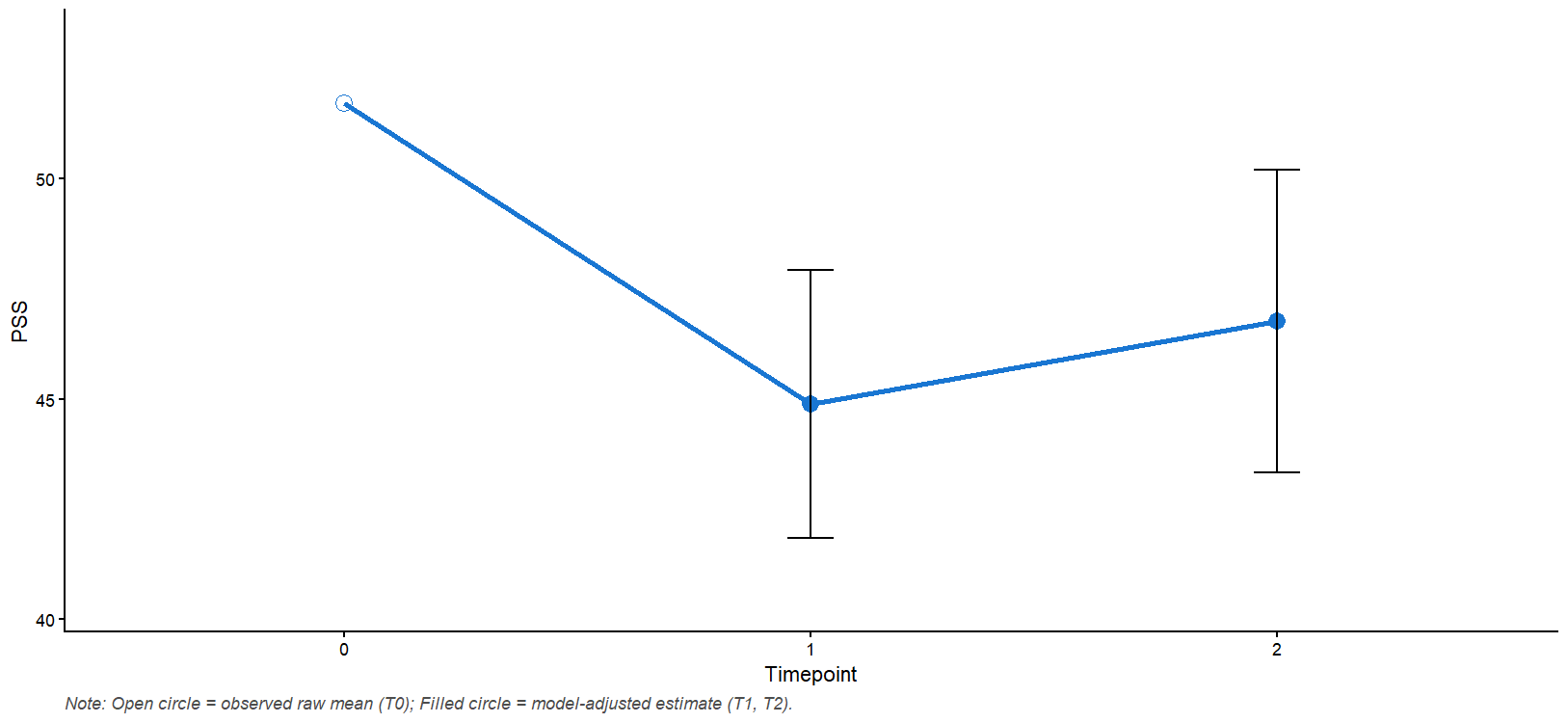


**Fig. S2**: PSS-10 scores at each time point. Note: vertical bars = 95% CIs.

**
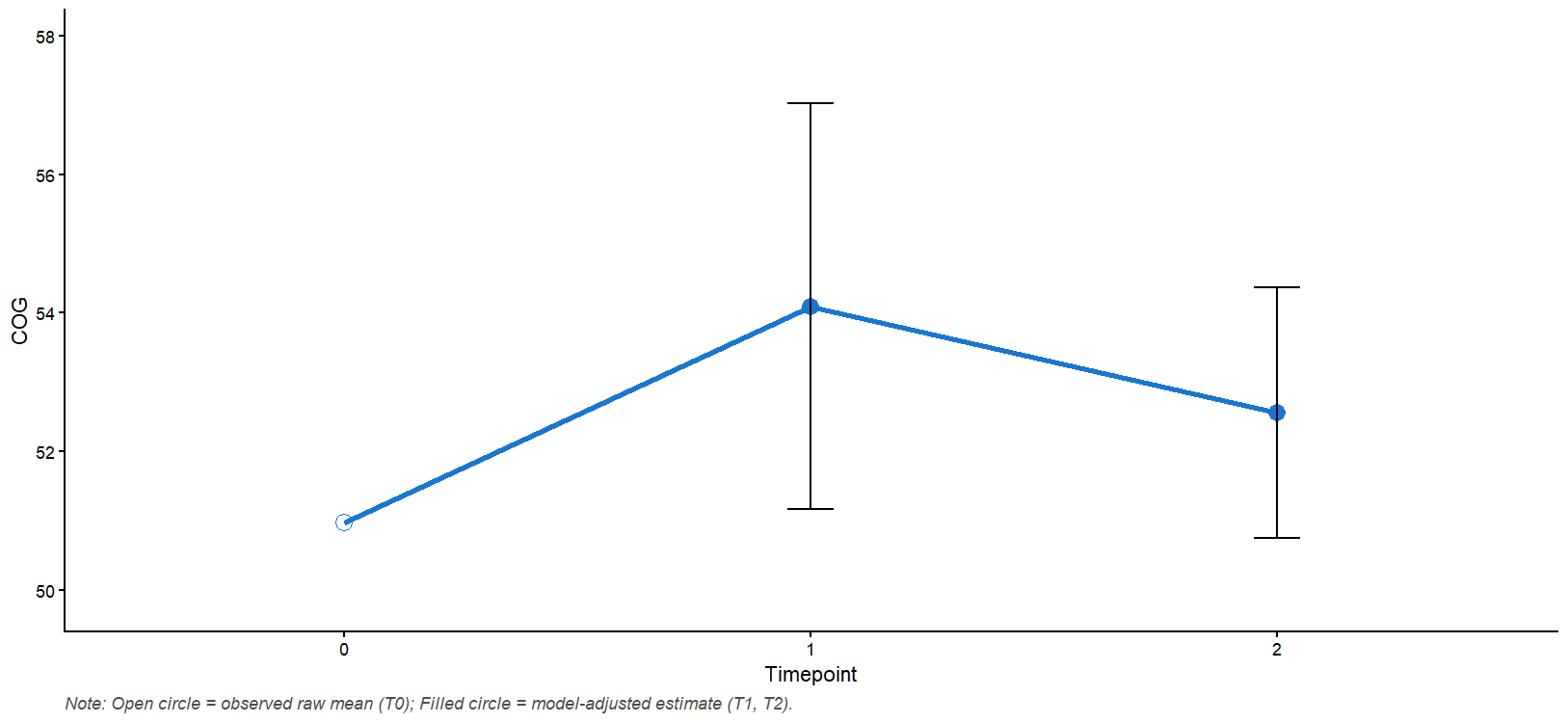
**
**Fig. S3:** PROMIS Cognition-8 scores at each time point. Note: vertical bars = 95% CIs


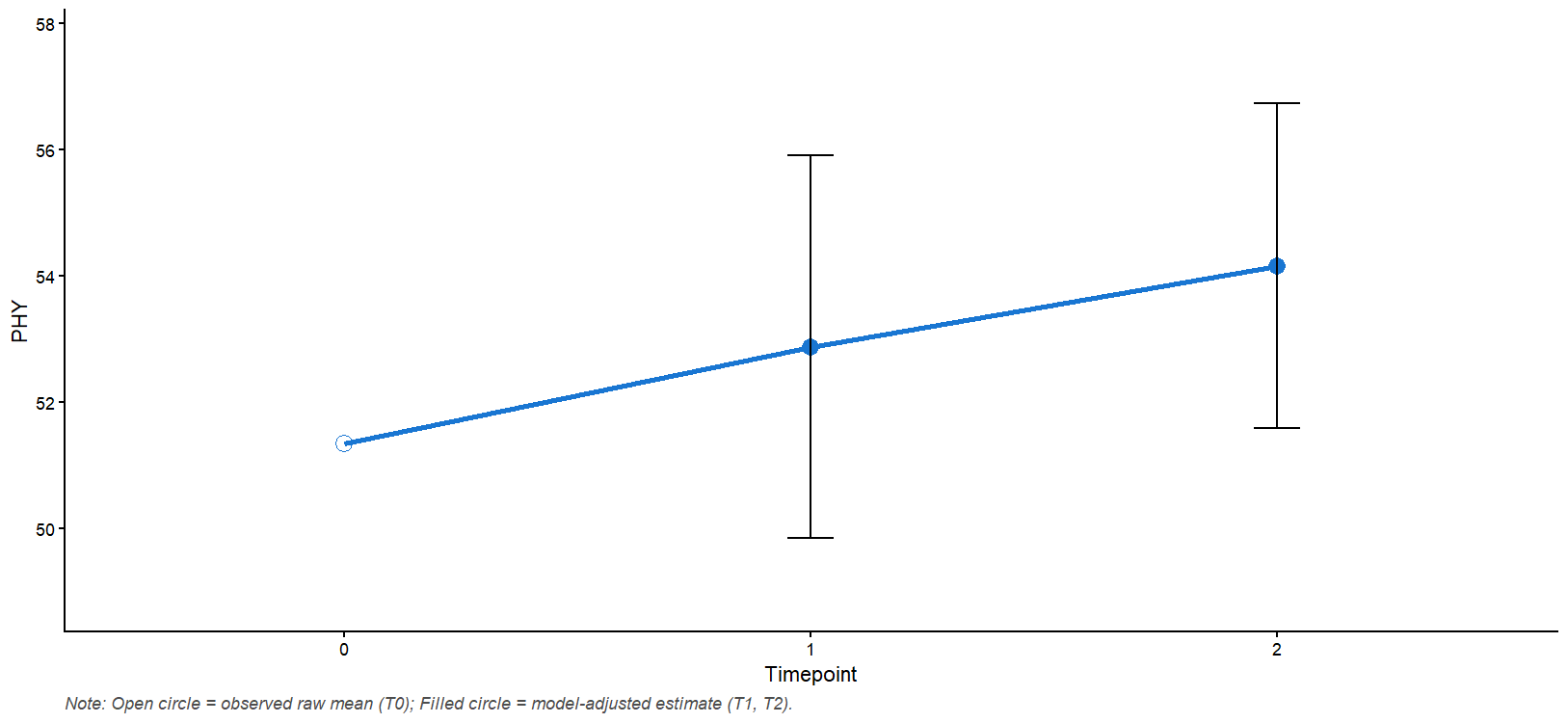


**Fig. S4**: PROMIS-29 Physical Function scores at each time point. Note: vertical bars = 95% CIs

**
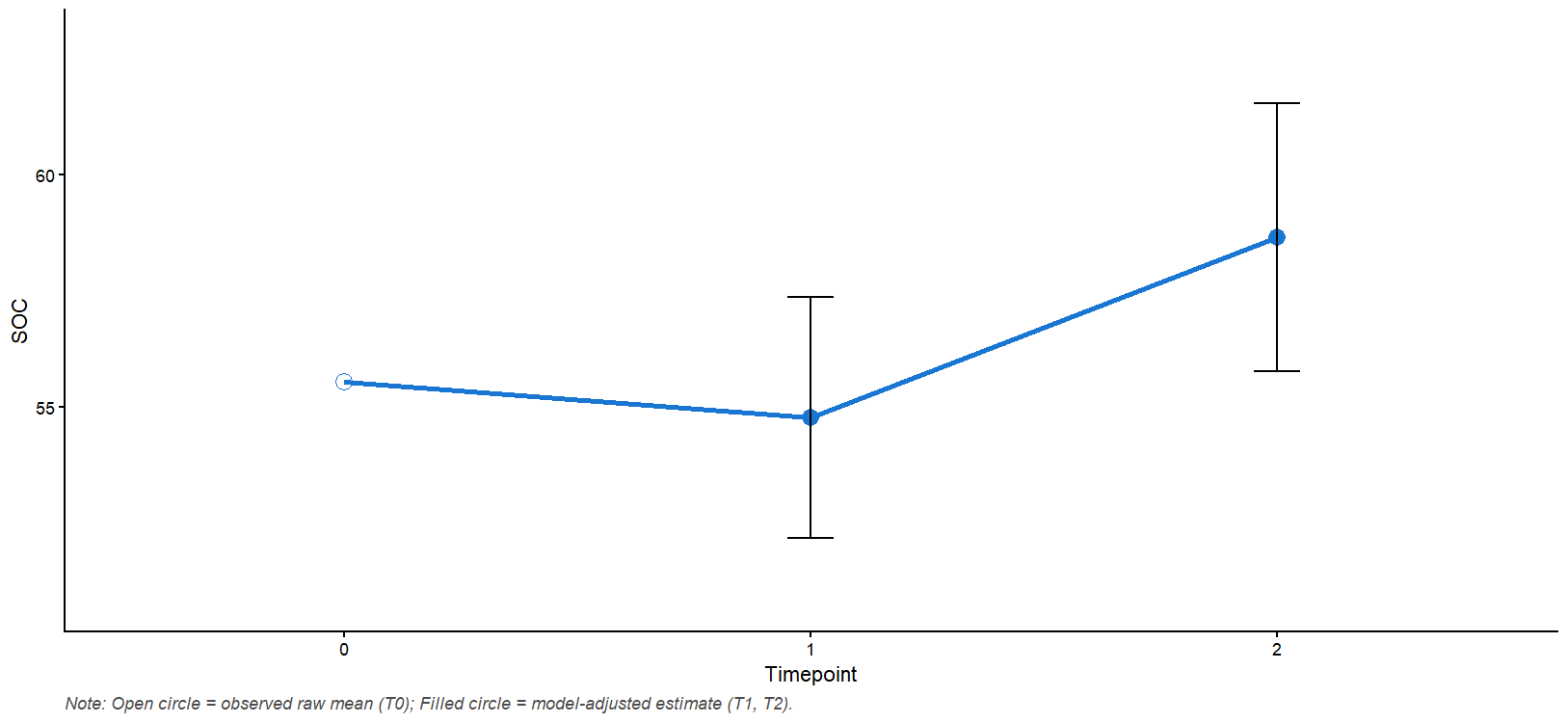
**
**Fig. S5**: PROMIS-29 Social Participation scores at each time point. Note: vertical bars = 95% CIs

**
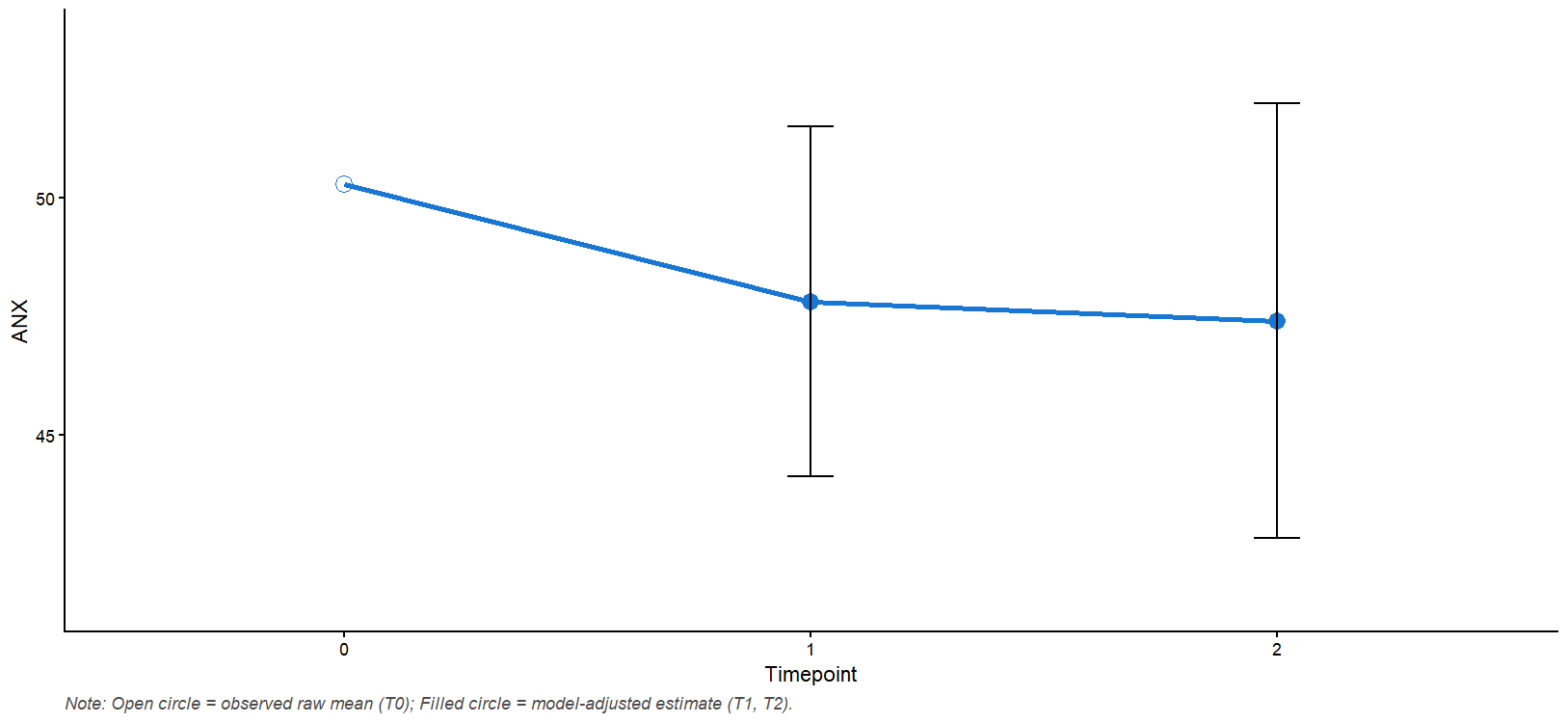
**
**Fig. S6**: PROMIS-29 Anxiety scores at each time point. Note: vertical bars = 95% CIs

**
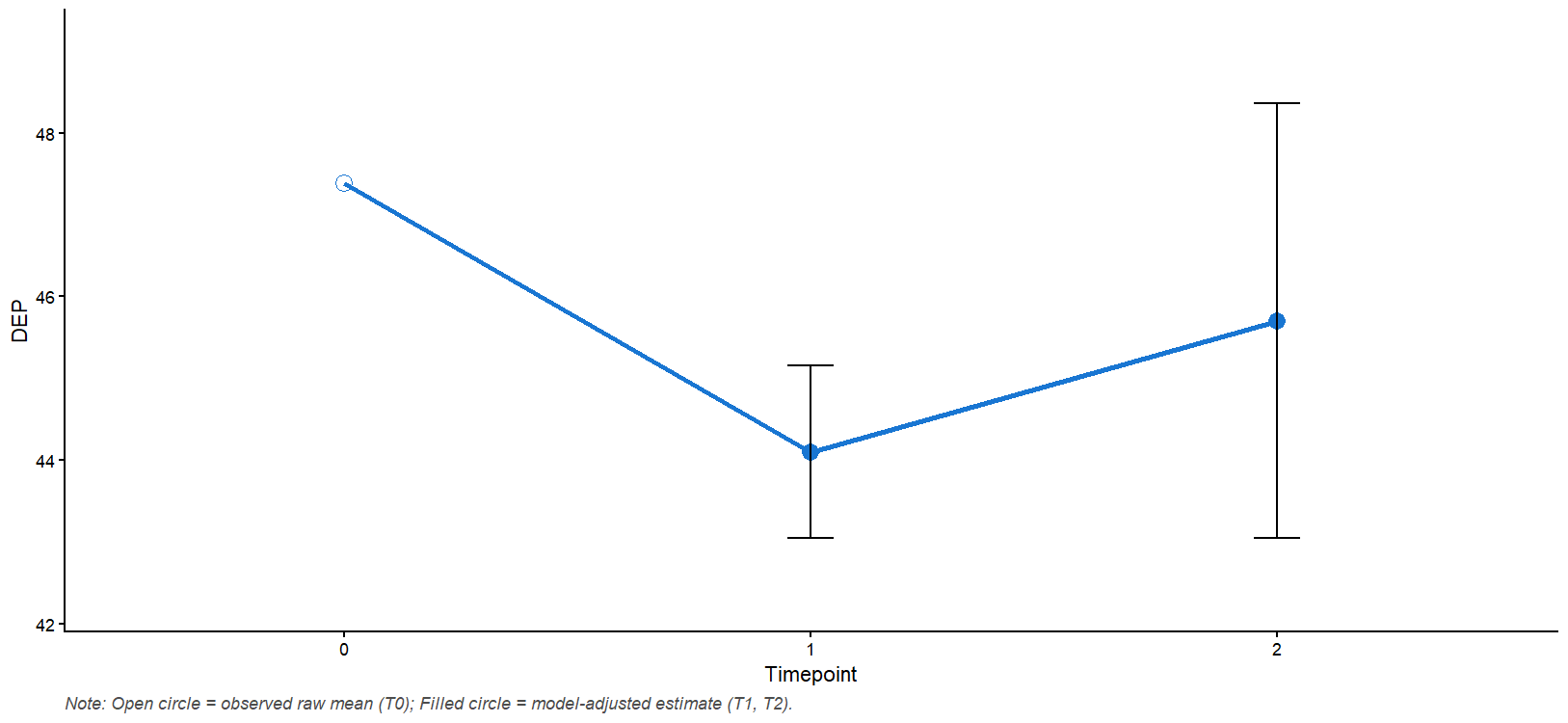
**
**Fig. S7**: PROMIS-29 Depression scores at each time point. Note: vertical bars = 95% CIs

**
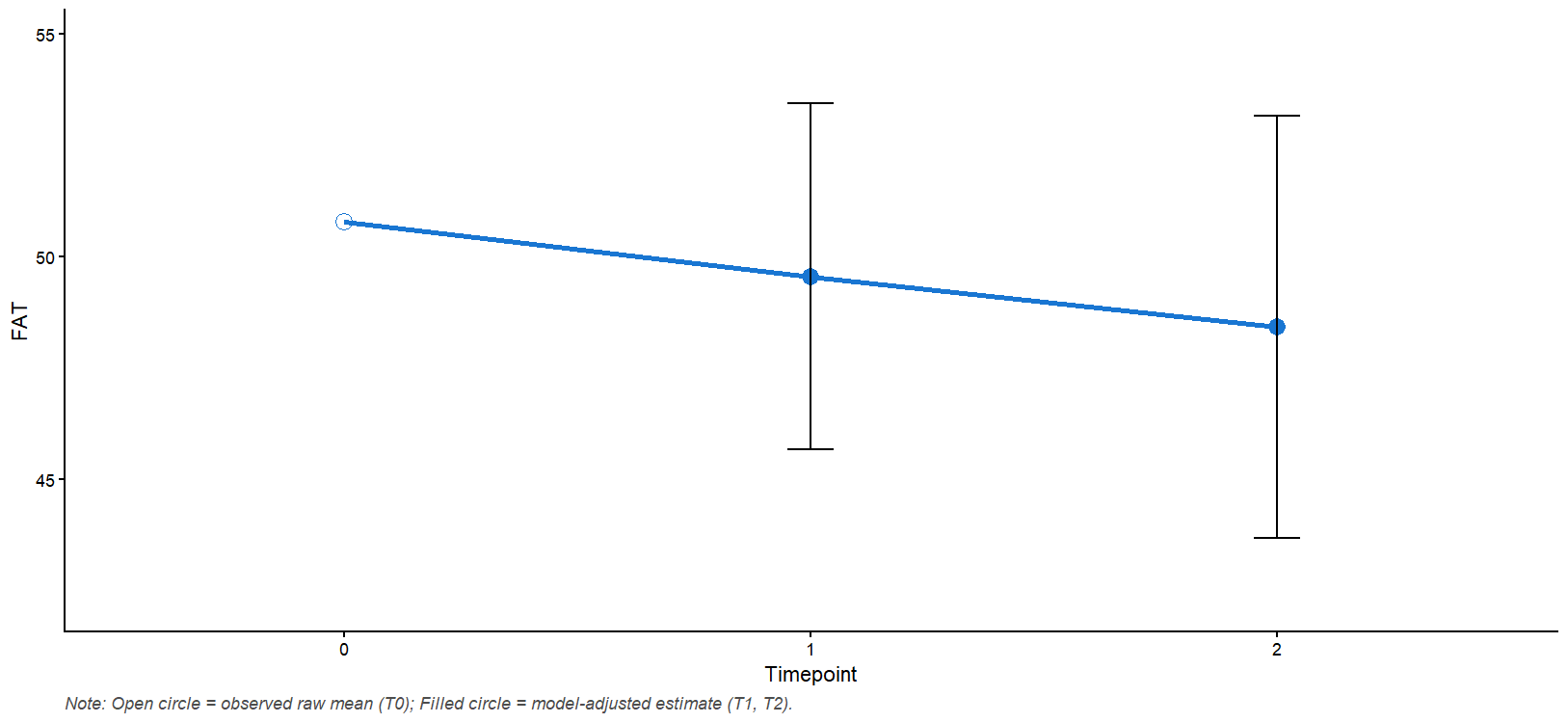
**
**Fig. S8**: PROMIS-29 Fatigue scores at each time point. Note: vertical bars = 95% CIs

**
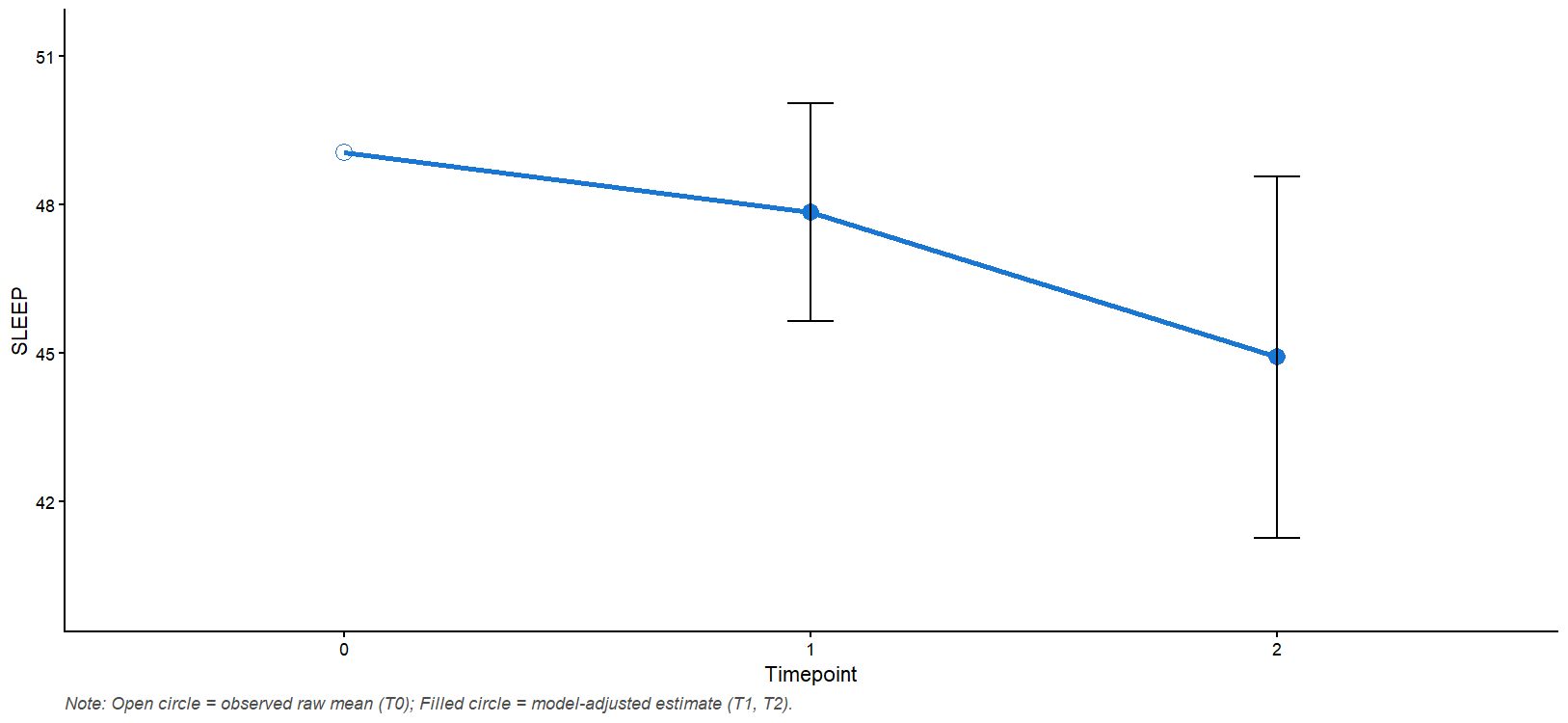
**
**Fig. S9**: PROMIS-29 Sleep Disturbance scores at each time point. Note: vertical bars = 95% CIs

**
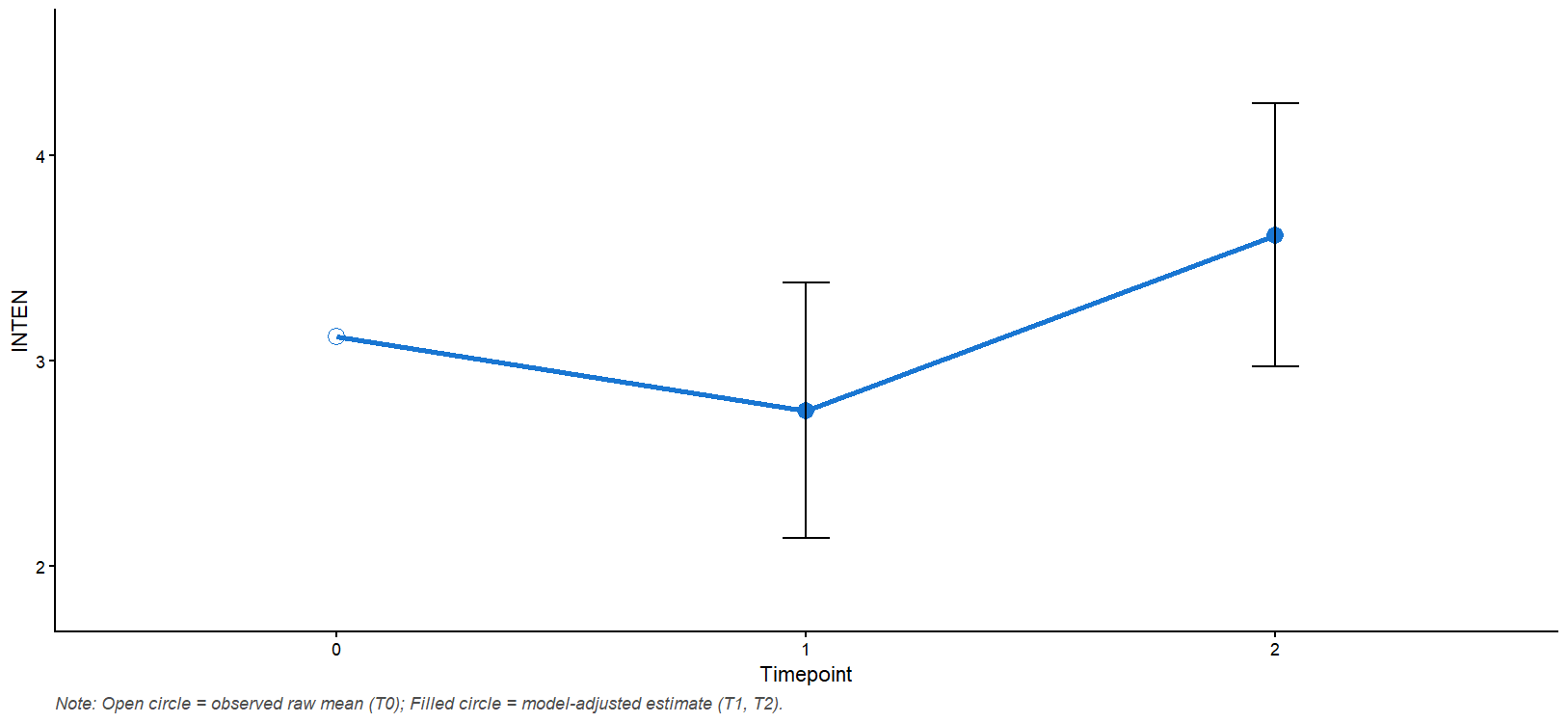
**
**Fig. S10**: PROMIS-29 Pain Intensity scores at each time point. Note: vertical bars = 95% CIs
